# Robust genome editing in adult vascular endothelium by nanoparticle delivery of CRISPR-Cas9 plasmid DNA

**DOI:** 10.1016/j.celrep.2021.110196

**Published:** 2022-01-04

**Authors:** Xianming Zhang, Hua Jin, Xiaojia Huang, Birendra Chaurasiya, Daoyin Dong, Thomas P. Shanley, You-Yang Zhao

**Affiliations:** 1 Program for Lung and Vascular Biology, Stanley Manne Children’s Research Institute, Ann & Robert H. Lurie Children’s Hospital of Chicago, Chicago, IL 60611, USA; 2 Department of Pediatrics, Division of Critical Care, Northwestern University Feinberg School of Medicine, Chicago, IL 60611, USA; 3 Ann & Robert H. Lurie Children’s Hospital of Chicago, Chicago, IL 60611, USA; 4 Department of Pharmacology, Northwestern University Feinberg School of Medicine, Chicago, IL 60611, USA; 5 Department of Medicine, Division of Pulmonary and Critical Care Medicine, Northwestern University Feinberg School of Medicine, Chicago, IL 60611, USA; 6 Feinberg Cardiovascular Research Institute, Northwestern University Feinberg School of Medicine, Chicago, IL 60611, USA; 7 Present address: Guangdong Provincial Key Laboratory of Medical Molecular Diagnostics, Guangdong Medical University College of Pharmacy, Dongguan, Guangdong, China; 8 Present address: Institute of Biomedical Engineering and Health Sciences, Changzhou University, Changzhou, Jiangsu, China; 9 These authors contributed equally; 10 Lead contact

## Abstract

Vascular endothelium plays a crucial role in vascular homeostasis and tissue fluid balance. To target endothelium for robust genome editing, we developed poly(ethylene glycol) methyl ether-*block*-poly(lactide-co-glycolide) (PEG-*b*-PLGA) copolymer-based nanoparticle formulated with polyethyleneimine. A single i.v. administration of mixture of nanoparticles and plasmid DNA expressing Cas9 controlled by *CDH5* promoter and guide RNA (*U6* promoter) induced highly efficient genome editing in endothelial cells (ECs) of the vasculatures, including lung, heart, aorta, and peripheral vessels in adult mice. Western blotting and immunofluorescent staining demonstrated an ~80% decrease of protein expression selectively in ECs, resulting in a phenotype similar to that of genetic knockout mice. Nanoparticle delivery of plasmid DNA could induce genome editing of two genes or genome editing and transgene expression in ECs simultaneously. Thus, nanoparticle delivery of plasmid DNA is a powerful tool to rapidly and efficiently alter expression of gene(s) in ECs for cardiovascular research and potential gene therapy.

## In brief

The ability to induce genome editing of the vascular endothelium has been challenging. Zhang et al. show that PP/PEI nanoparticles could deliver plasmid DNA to adult mice for robust genome editing and 80% knockdown of protein expression selectively in ECs of various vascular beds and also for transgene expression simultaneously.

## Graphical abstract

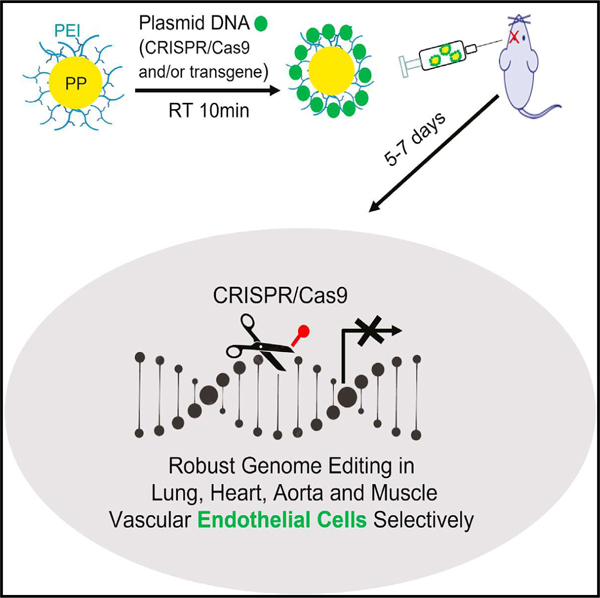

## INTRODUCTION

The vascular endothelium is a monolayer of endothelial cells (ECs) lining the luminal surface of blood vessels. The endothelial monolayer plays a crucial role in vascular homeostasis and maintenance of tissue fluid balance (Aird, 2008; Carmeliet, 2000; Cines et al., 1998; Rafii et al., 2016). It helps to maintain an anti-thrombotic and anti-inflammatory state of the microvascular bed, and control the tone and proliferative state of the underlying vascular smooth muscle cells (Aird, 2008; Cines et al., 1998; Owens et al., 2004). ECs also mediate diverse biological functions, such as endocytosis and metabolism, and directing organ regeneration and repair (Carmeliet, 2000; Ghesquiere et al., 2014; Rafii et al., 2016). Under adverse conditions (as for example, infection, tissue necrosis, immune reactions, or hypercholesterolemia), ECs are activated, leading to inflammation and endothelial barrier disruption (increased vascular permeability, edema formation, release of proinflammatory cytokines, and leukocyte extravasation) (Pober and Sessa, 2007). Endothelial dysfunction figures prominently in the etiologies of many diseases such as atherosclerosis, the pathological process underlying the major cardiovascular diseases (myocardial infarction, stroke, coronary artery disease, and peripheral artery disease) (Hansson, 2005; Libby et al., 2002; Ross, 1999), sepsis, acute respiratory distress syndrome (Aird, 2003; Lee and Slutsky, 2010; Matthay et al., 2012), and COVID-19 respiratory distress (Ackermann et al., 2020; Marini and Gattinoni, 2020; Varga et al., 2020).

The clustered regularly interspaced short palindromic repeats (CRISPR)-Cas9 system is extensively employed to edit the genome of zygotes for generation of various genetically modified animal species, including mice, rats, and even monkeys and to explore the application of CRISPR-Cas9 in postnatal or adult animals (Amoasii et al., 2018; Cong et al., 2013; Doudna and Charpentier, 2014; Mali et al., 2013; Nelson et al., 2016; Tabebordbar et al., 2016). CRISPR-Cas9 holds great promise for the treatment of human disease, especially genetic disease by correcting disease-causing or associated mutations in the genome. There is a great interest in developing safe and efficient therapeutic tools to deliver the CRISPR-Cas9 system to the body to target the specific organ and cell type (Cox et al., 2015; Varshney et al., 2015). One of the delivery systems with good *in vivo* genome editing efficacy is the recombinant adeno-associated virus (AAV) (Amoasii et al., 2018; Nelson et al., 2016; Ran et al., 2015; Schmidt and Grimm, 2015; Tabebordbar et al., 2016), but this approach is potentially problematical for several reasons. The viral vector is capable of triggering high immune response and has a low packaging size (restricted to 4.7 kb in AAVs) (Cox et al., 2015; Ertl, 2017; Mingozzi and High, 2013; Varshney et al., 2015). Extended expression of Cas9 mediated by viral vector may cause unwanted DNA damage and immunogenicity. For these reasons, the use of a nonviral vector has attracted widespread interest. Efficient genome editing was obtained in the liver with lipid nanoparticles but remains a challenge for organs besides the liver (Finn et al., 2018; Yin et al., 2016, 2017). A lipid-modified polymer nanoparticle delivery system was recently developed to target blood cells (e.g., macrophages) showing considerably low genome editing rate (20%) in mice (Luo et al., 2018). The ability to induce robust genome editing of the vascular endothelium has been challenging thus far, considering the lack of a delivery system capable of targeting the vascular endothelium other than the liver and eye.

In the present study, we describe the development of poly(ethylene glycol) methyl ether-*block*-poly(lactide-co-glycolide) (PEG-*b*-PLGA; PP)-based nanoparticles with excellent biodistribution for vascular delivery and show that polyethyleneimine (PEI)-formulated PP nanoparticle-mediated delivery of the all-in-one-CRISPR plasmid DNA expressing Cas9 under the control of human *CDH5* promoter and guide RNA (gRNA) driven by the *U6* promoter results in highly efficient genome editing specifically in ECs of various vascular beds including lung, heart, aorta, and peripheral vasculature in adult mice with a single administration, which leads to disruption of gene expression (~80% decrease of protein expression in ECs) and a phenotype mimicking that of genetic knockout mice. PP/PEI nanoparticle delivery of plasmid DNA could induce genome editing of at least two genes or introduce genome editing and transgene expression in ECs simultaneously. These data demonstrate that nanoparticle delivery of CRISPR plasmid DNA is a simple powerful tool to rapidly and efficiently alter expression of gene(s) in vascular endothelium. This provides a significant advance in cardiovascular research to facilitate delineation of molecular mechanisms and identify druggable targets for vascular diseases.

## RESULTS

### Development and characterization of PP/PEI nanoparticles for better biodistribution

**Figure 1.**
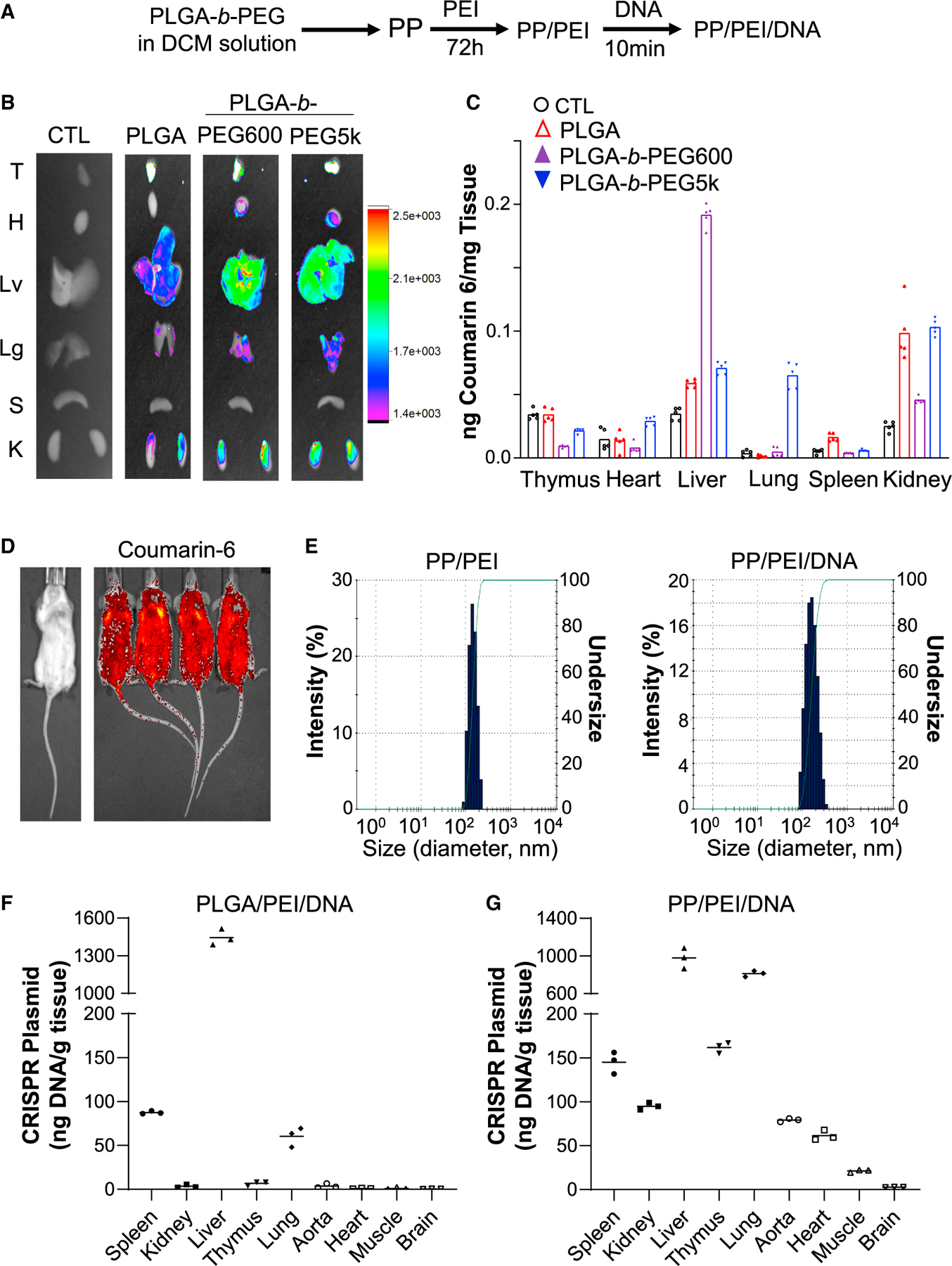
Characterization and biodistribution of the PLGA-based nanoparticles (A) Diagram showing the procedures to generate PP/PEI nanoparticle for plasmid DNA delivery. (B) Superior biodistribution of PEG_5000Da_-*b*-PLGA (PP) nanoparticles. IVIS imaging of fluorescent dye coumarin 6-loaded nanoparticles in various organs at 8 h post-retro-orbital administration. CTL, naïve mice without nanoparticle administration. T, thymus; H, heart; Lv, liver; Lg, lung; S, spleen; K, kidney. (C) Quantification of biodistribution by normalizing the fluorescent intensity to the tissue weight (n = 5). Bar box represents mean. (D) Fluorescent tomography by IVIS imaging of live mice demonstrating whole body distribution of the PP nanoparticles. The image was taken 5 h after injection of coumarin 6-loaded PP nanoparticles (no plasmid DNA) via the retro-orbital venous plexus. (E) Size distribution of PP/PEI nanoparticles and PP/PEI/DNA complex. The size distribution of the nanoparticles was characterized with a Zetasizer. The study was repeated four times with similar data. (F and G) Quantification of CRISPR plasmid DNA in various organs after nanoparticle delivery. The CRISPR plasmid DNA (40 μg/mouse) was delivered into 3- to 4-month-old mice by PLGA/PEI (without PEG) (F) or PP/PEI (G) nanoparticles through retro-orbital injection. The amount of Cas9 cDNA carried in the plasmid DNA at 8 h post-administration was determined by quantitative PCR (QPCR) analysis, calculated using a standard curve generated from the CRISPR plasmid DNA, and normalized to the tissue weight. n = 3 (F, G). Bars represent means.

To develop an efficient cardiovascular delivery system, we employed the biodegradable material PLGA or PEG-*b*-PLGA copolymer with different molecular weight of PEG as a starting material to generate nanoparticles (Figure 1A). As shown in Figures 1B and 1C, nanoparticles made with PLGA alone or PLGA-PEG_600Da_ were highly enriched in mouse liver with little in heart and lung at 8 h post-retro-orbital administration. PEG_5000 Da_-*b*-PLGA nanoparticles achieved best tissue distribution in heart and lung. Thus, we generated PEG_5000_-*b*-PLGA (designated as PP) nanoparticles with a goal for whole vascular system delivery. PP nanoparticles exhibited a quite even whole-body distribution without specific enrichment in the liver at 5 h post-retro-orbital administration as revealed by IVIS tomography (Figure 1D). Given the excellent nucleic acid condensation capacity of large molecular weight PEI, the PP nanoparticles were incubated with PEI_25,000 Da_ to formulate PP/PEI nanoparticles for DNA delivery. PP/PEI nanoparticles also exhibited good tissue distribution in lung, aorta, and heart (Figure S1). Zetasizer analysis showed the size of the PP/PEI nanoparticles were 80 to 250 nm in diameter (Figure 1E). The size of the PP/PEI nanoparticles complexed with the all-in-one CRISPR^*CAG*^ plasmid DNA (Ran et al., 2013) (Figure S2A) expressing Cas9 under the control of the chicken *Actb* promoter with a CMV enhancer (*CAG*) and gRNAs driven by the *U6* promoter were less than 300 nm with 90% of them in the range of 80 to 250 nm (Figure 1E).

At 8 h after retro-orbital administration, CRISPR plasmid DNA delivered by PLGA/PEI (without PEG) nanoparticles was highly enriched in liver with only small amount (~1/20th of the liver level) in spleen and lung; there was no detectable DNA in heart, aorta, and skeletal muscle (Figure 1F). In contrast, PP/PEI nanoparticle delivery resulted in broad tissue distribution of CRISPR plasmid DNA. Liver and lung had the highest accumulation, but other organs such as thymus, spleen, kidney, heart, aorta, and skeletal muscle also had DNA accumulation (Figure 1G), demonstrating the PP/PEI nanoparticles can deliver plasmid DNA to all the major organs. Then, we assessed the kinetics of PP/PEI/plasmid DNA in various organs. At 48h post-retro-orbital administration, approximately 50% of the plasmid DNA levels at 8 h was accumulated in most of the organs except kidney and thymus (Figure S2B). From 10% to 20% of the 8-h plasmid DNA levels remained in lung, heart, aorta, and skeletal muscle at 96 h post-administration.

### CRISPR^CDH5^ plasmid DNA dose responses of *in vivo* genome editing

To identify a positive gRNA for *in vivo* use, we employed the nanoparticles for *in vitro* DNA transfection in cultured cell line. The PP/PEI nanoparticles bound to CRISPR plasmid DNA efficiently neutralized its negative charge and resulted in 90% transfection efficiency in Hepa-1c1c7 cells (Figure S3). Three days following transfection, cells were lysed for genomic DNA isolation and insertion/deletion (indel) mutation analysis. Sanger sequencing decomposition analysis using TIDE software (Brinkman et al., 2014) revealed that gRNA1 targeting the mouse *Pik3cg* gene, which encodes the p110γ isoform of PI3K, caused 80% genome editing in cultured cells, whereas other gRNAs induced less than 20% genome editing (Figure S3C).

**Figure 2.**
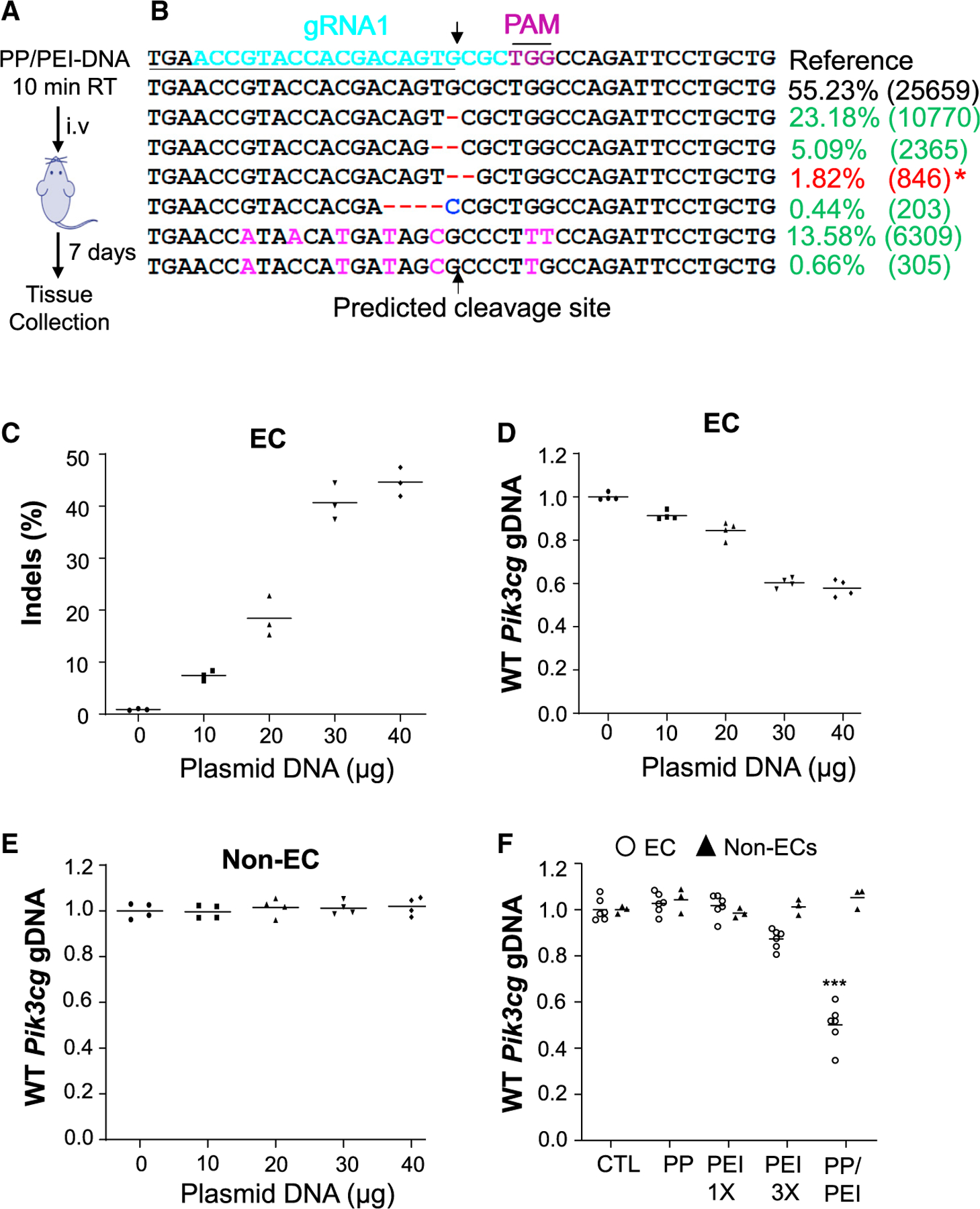
Dose response of CRISPR^*CDH5*^ plasmid DNA in inducing EC-specific genome editing in adult mice (A) Schematic presentation of nanoparticle-mediated delivery of the CRISPR system to adult mice. After 10-min incubation at RT, the complex was administered i.v. (retro-orbitally). (B and C) Next generation sequencing analysis demonstrating robust genome editing in lung ECs. Representative next generation sequencing analysis demonstrating various insertion and deletion mutations in lung ECs of a mouse administered with 40 μg plasmid DNA (B). The underlined sequence is the forward primer used for QPCR analysis of genome editing. * indicates the deletion mutation happened to result in a perfect 3^′^ match with the forward primer, thus it can be amplified by PCR. The indels of the 50-bp region targeted by the gRNA1 was quantified following next generation sequencing (C) (n = 3). (D) QPCR analysis of genome editing efficiency in lung ECs. The reduced amount of wild-type (WT) genomic DNA in the targeted region indicates DNA mutations that failed to be amplified due to 3^′^ mismatch (n = 4). (E) QPCR analysis demonstrating no genome editing in non-ECs in mouse lungs (n = 4). (F) QPCR analysis demonstrating that only PP/PEI nanoparticle delivery of CRISPR^*CDH5*^ plasmid DNA could induce robust genome editing in mouse lung ECs; 40 μg of plasmid DNA was mixed with PP/PEI nanoparticles, PP nanoparticles, PEI 13, or PEI 33 amount of PEI contained in the PP/PEI nanoparticles and administered retro-orbitally to mice (n = 6, ECs; n = 3, non-ECs). Bars represent means. ***p < 0.001 versus CTL (Control) ECs. Student’s t test.

We next addressed the possibility that PP/PEI nanoparticles deliver the CRISPR plasmid DNA to the whole cardiovascular system and thereby induce efficient *in vivo* genome editing. To specifically express Cas9 in ECs, we replaced the *CAG* promoter in the CRISPR^*CAG*^ plasmid DNA with the 3.5 kb human *CDH5* promoter which is EC-specific (Gory et al., 1999; Huang et al., 2016; Prandini et al., 2005). The PP/PEI nanoparticles complexed with CRISPR^*CDH5*^ plasmid DNA expressing gRNA1 were administered to adult mice via retro-orbital injection (Figure 2A). To determine a plasmid DNA dose for effective genome editing in vascular ECs, we carried out a dose-response study. At 7 days post-administration, the mice were euthanized for tissue collection, and ECs and non-ECs were then isolated. As shown in Figures 2B and 2C, 40 μg of CRISPR^*CDH5*^ plasmid DNA/adult mouse induced a 45% indel efficiency in lung ECs revealed by next generation sequencing analysis.

### Validation of QPCR analysis as a simple and reliable method for detection of indels

The indels are generated through the deletion or insertion of base(s) during nonhomologous end-joining (NHEJ) repair after Cas9 cleavage-induced DNA double-strand breaks (Ran et al., 2015). Given that the frequency of indels formed by base deletion is 3- to 4-fold greater than by base insertion, and that the deleted bases are often located upstream of the Cas 9 cleavage site (Cox et al., 2015; Varshney et al., 2015), we designed a pair of PCR primers with the forward primer 3′ ended with the predicted cleavage site for quantitative PCR (QPCR) analysis of indels, as the forward primer would not amplify the mutated DNA with deletions and insertions near the predicted cleavage site due to a 3′ mismatch but amplify the wild-type DNA (Figures S4A and 2B). As shown in Figure 2B, we expected the forward primer would not amply the genomic DNA with all the mutations except one kind of deletion mutation, which happened to have a perfect 3′ match and thus 43.0% indel efficiency. QPCR analysis demonstrates a 42.2% indel efficiency in lung ECs (Figure 2D). These data are consistent with the data from next generation sequencing analysis.

To further validate the QPCR method for quantitative detection of single base pair indels, we made plasmid DNAs with single base pair deletion or insertion and designed primers specific to the 3′ wild-type or insertion base pair (Figure S4B). As shown in Figures S4C–S4E, the wild-type primer was not able to amplify the mutant plasmid DNA with single base pair deletion. Similarly, the primer specific to the 3′ single nucleotide insertion could not amplify the wild-type DNA (Figures S4F–S4H). Thus, the QPCR-based genomic DNA analysis is a relatively simple and reliable method for indel quantification. QPCR analysis also revealed similar indel rates in mice administered with 50 μg plasmid DNA (data not shown), demonstrating 40 μg plasmid DNA/mouse is the optimal dose to achieve highest genome editing *in vivo*. QPCR analysis revealed no indels in non-ECs, confirming the EC-specific genome editing *in vivo* (Figure 2E).

### Robust genome editing in lung ECs with PP/PEI nanoparticle delivery of plasmid DNA

To compare the delivery efficiency of various nanoparticles and PEI_25,000 Da_, the same amount of CRISPR^*CDH5*^ plasmid DNA expressing gRNA1 (40 μg/adult mouse) was complexed with PP nanoparticles, PP/PEI nanoparticles, or PEI_25,000_ equivalent to 13 or 33 of the PEI amount used in the PP/PEI nanoparticles and administered to different cohorts of mice. At 7 days post-administration, lung tissue was collected for EC and non-EC isolation. QPCR analysis demonstrated that wild-type *Pik3cg* genomic DNA was decreased by ~50% in ECs isolated from lungs of PP/PEI/gRNA1 plasmid-transduced mice compared with control naïve mice (Figure 2F). However, neither PP nanoparticle nor 13 PEI delivery induced genome editing in lung ECs, although 33 PEI delivery resulted in approximately 10% genome editing in ECs. Thus, the PP/PEI nanoparticle is a powerful delivery tool to achieve robust *in vivo* genome editing in vascular endothelium in adult mice.

**Figure 3.**
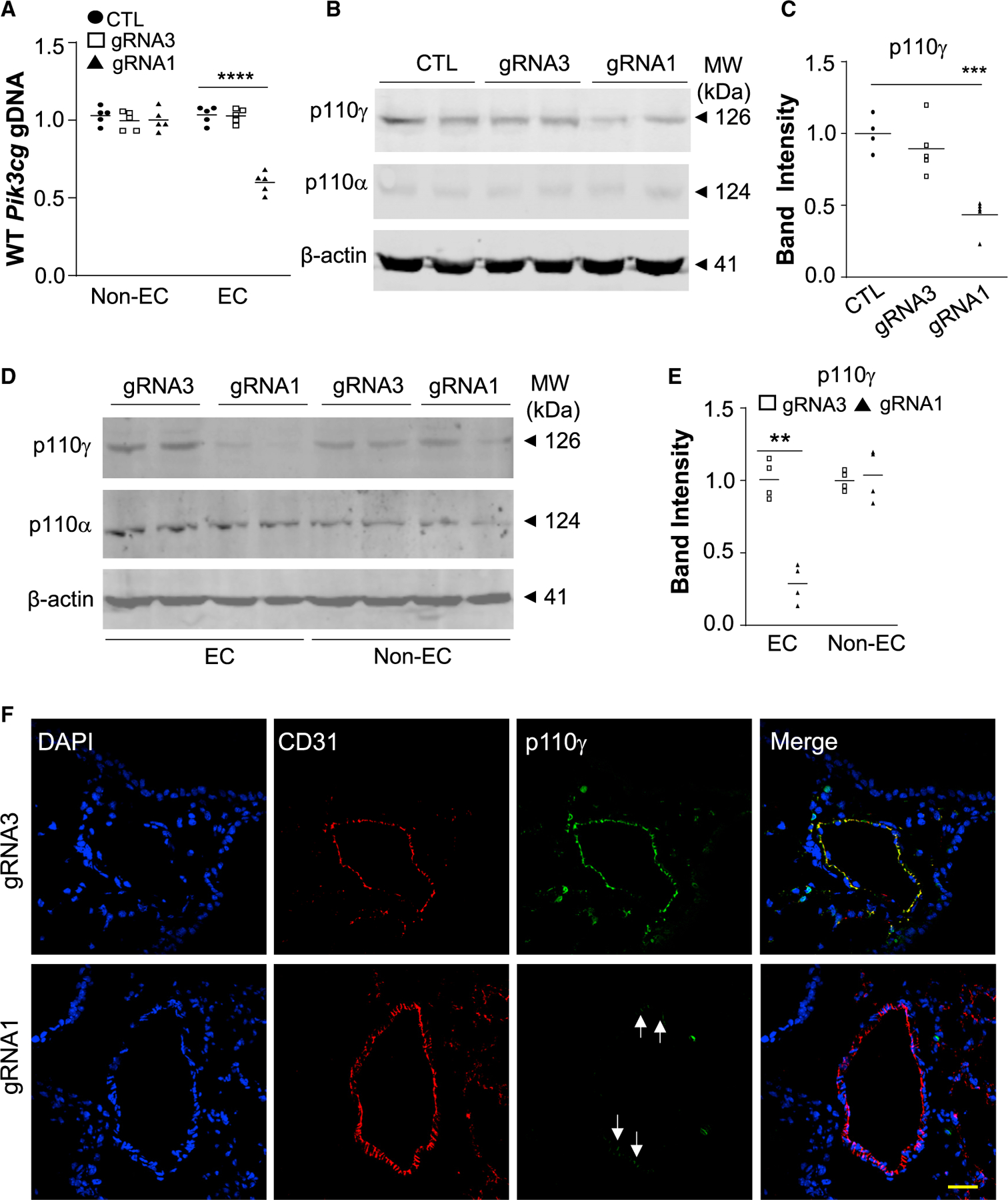
Robust genome editing in pulmonary vascular ECs leading to diminished protein expression in ECs (A) QPCR analysis demonstrating robust EC-specific genome editing in mouse lungs by *Pik3cg* gRNA1. Forty micrograms of CRISPR^*CDH5*^ plasmid DNA expressing either gRNA1 or gRNA3 was delivered to adult mice, respectively. Mice without plasmid DNA delivery (CTL) were used as comparison. Although gRNA3 could induce 20% indels in cultured cells, it was not effective in inducing genomic editing in lung ECs *in vivo* (n = 3). (B) Representative western blotting demonstrating a marked decrease of p110γPI3K protein expression in lung tissues of CRISPR^*CDH5*^/gRNA1 nanoparticle-transduced mice. Expression of p110αPI3K was not affected, demonstrating gene-specific disruption. (C) Quantification was carried out with Image J (n = 4, 5, 5). (D and E) Western blotting demonstrating diminished p110γPI3K protein expression in ECs isolated from lungs of CRISPR^*CDH5*^/gRNA1 nanoparticle-treated mice (n = 4). (F) Representative micrographs of immunofluorescent staining showing diminished p110γPI3K expression in pulmonary vascular ECs of CRISPR^*CDH5*^/gRNA1 nanoparticle-treated mice. Cryosections were immunostained with anti-CD31 (marker for ECs) (red) and anti-p110γPI3K (green). Nuclei were counterstained with DAPI (blue). Arrows point to ECs with less efficient knockdown. ***p < 0.001, Student’s t test (A, E); one-way ANOVA with Bonferroni post hoc multiple analysis (C). Scale bar, 50 μm.

Employing the PP/PEI nanoparticles to deliver the CRISPR^*CDH5*^ plasmid DNA to wild-type mice, we observed 40% genome editing efficiency in lung ECs but not in non-ECs (Figure 3A) in mice transduced with gRNA1 plasmid DNA. Western blotting demonstrated 55% decrease of p110γPI3K protein expression in lungs of gRNA1 plasmid-transduced mice compared with control mice (Figures 3B and 3C), and more than 70% (equivalent to 80% given the cell purity of 90%) decrease of p110γPI3K protein expression in lung ECs but not non-ECs of gRNA1 plasmid-transduced mice compared with gRNA3 plasmid-transduced mice (Figures 3D and 3E). However, expression of the p110α isoform of PI3K was not affected (Figure 3D), demonstrating gene-specific disruption of expression. Immunofluorescent staining also revealed diminished p110γPI3K expression in pulmonary vascular ECs in gRNA1 but not gRNA3 plasmid-transduced mice (Figure 3F). Together, these data demonstrate highly efficient EC- and gene-specific genome editing in adult mice following a single administration of PP/PEI nanoparticle:CRISPR plasmid DNA mixture.

We also determined if nanoparticle delivery of the CRISPR^*CDH5*^ plasmid DNA under pathological condition is also efficient to induce genome editing. We challenged wild-type mice with endotoxin lipopolysaccharide (LPS) i.p., which induces sepsis and inflammatory lung injury. Twenty hours later, the complex of PP/PEI nanoparticles:CRISPR^*CDH5*^ plasmid DNA expressing gRNA1 was administered retro-orbitally and lung tissues were collected at 7 days post-delivery for genome editing analysis. As shown in Figures 5A–5C, post-injury delivery of CRISPR^*CDH5*^ plasmid DNA induced ~45% genome editing efficiency selectively in lung ECs.

### Robust genome editing in ECs leads to a phenotype seen in genetic knockout mice

**Figure 4.**
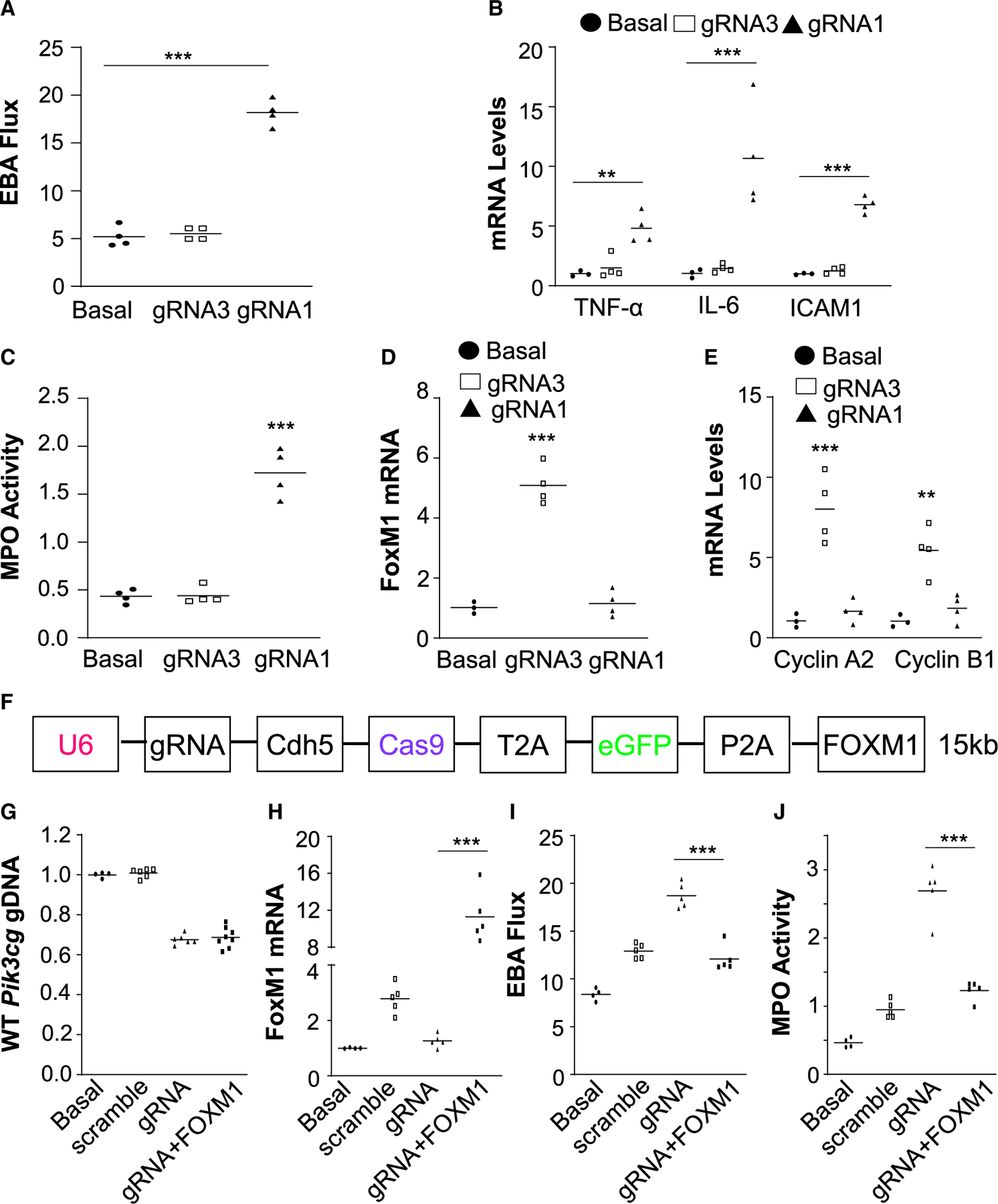
Genome editing-induced disruption of p110γPI3K expression in pulmonary vascular ECs resulted in impaired vascular repair and resolution of inflammation as seen in *Pik3cg*^−/−^ mice (A) Measurement of pulmonary transvascular EBA flux demonstrating defective vascular repair in gRNA1 nanoparticle-treated mice in contrast to gRNA3 nanoparticle-treated mice. Seven days post-nanoparticle:DNA administration, the mice were challenged with LPS. At 72 h post-LPS, lung tissues were collected for analyses (n = 4). Basal, WT mice without administration of nanoparticle/DNA and LPS challenge. (B) QRT-PCR analysis demonstrating marked increases of expression of proinflammatory genes in lungs of gRNA1 nanoparticle-treated mice (n = 3 or 4). (C) Persistently elevated lung myeloperoxidase (MPO) activity in gRNA1 nanoparticle-treated mice (n = 4). (D and E) QRT-PCR analysis showing inhibited expression of the transcription factor FoxM1 (D) and its target genes (E) in lungs of gRNA1 nanoparticle-treated mice (n = 3 or 4). (F) Diagram showing a large *CRISPR*^*CDH5*^ plasmid expressing a 3-kb *FOXM1* transgene. (G) Similar genome editing efficiency in lung ECs by different sizes of CRISPR plasmid. Four days post-administration of PP/PEI nanoparticles:plasmid DNA complexes (40 μg/mouse), the mice were challenged with LPS and lung tissues were collected at 72 h post-LPS for various analyses (n = 4 or 6). (H) QRT-PCR analysis showing 12-fold induction of FoxM1 expression in lungs of mice with the CRISPR^*CDH5*^-*FOXM1* plasmid (n = 4 or 5). (I and J) FOXM1 transgene expression recused the defective vascular repair phenotype induced by gRNA-mediated knockout of p110γPI3K, which is the upstream mediator (n = 4 or 5). **p < 0.01, ***p < 0.001. One-way ANOVA with Bonferroni post hoc analysis (A, C, D); Student’s t test (B, E, H, I, J).

Our published study showed that genetic deletion of *Pik3cg* in lung ECs interfered with vascular repair and resolution of inflammation following sepsis-induced inflammatory vascular injury (Huang et al., 2016). We next determined whether CRISPR-mediated genome editing of *Pik3cg* would result in a similar phenotype in wild-type adult mice. At 7 days after nanoparticle/CRISPR^*CDH5*^ plasmid DNA delivery, the mice were challenged with LPS to induce inflammatory vascular injury. At 72 h post-LPS challenge (when wild-type mice were fully recovered), lung tissues were collected for determination of vascular permeability and inflammation. Measurements of pulmonary transvascular flux of Evans blue-conjugated albumin (EBA; a measure of protein permeability) showed a markedly elevated EBA value at 72 h post-LPS in gRNA1 plasmid-transduced mice, whereas permeability recovered to the basal value in gRNA3 plasmid-transduced mice (Figure 4A), indicating impaired vascular repair in gRNA1 plasmid-transduced mice as seen in *Pik3cg*^−*/*−^ mice (Huang et al., 2016). Lung inflammation, evident by marked increases of expression of pro-inflammatory cytokines and of myeloperoxidase activity (indicative of neutrophil sequestration), was not resolved in gRNA1 plasmid-transduced mice at 72 h post-LPS in contrast to gRNA3 plasmid-transduced mice (Figures 4B and 4C), consistent with defective vascular repair in gRNA1 plasmid-transduced mice. Expression of the reparative transcription factor FoxM1, which is downstream of p110γPI3K signaling (Huang et al., 2016) and mediates EC proliferation and re-annealing of adherens junctions for vascular repair (Mirza et al., 2010; Zhao et al., 2006) during the recovery phase, was induced in gRNA3 plasmid-transduced mice as seen in wild-type mouse lungs but not in gRNA1 plasmid-transduced mice (Figure 4D), further demonstrating the inhibition of p110γPI3K signaling in gRNA1 plasmid-transduced mouse lungs. Accordingly, expression of the FoxM1 target genes *Ccna2* and *Ccnb1*, essential for cell cycle progression, was not induced in gRNA1 plasmid-transduced mouse lungs (Figure 4E). These data demonstrate that PP/PEI nanoparticle delivery of CRISPR^*CDH5*^ plasmid DNA induces highly efficient genome editing in lung ECs leading to diminished p110γPI3K expression, which in turn results in defective vascular repair and resolution of inflammation through inhibited FoxM1 expression as seen in *Pik3cg*^−*/*−^ mice (Huang et al., 2016).

### No detrimental effect of increased size of plasmid DNA on genome editing efficiency and phenotype rescue by dual FOXM1 transgene expression

We next determined if the size of the plasmid DNA affects indel efficiency. A *FOXM1* cDNA (3kb) was inserted into the CRISPR^*CDH5*^ plasmid DNA (Figure 4F). At 4 days after retro-orbital injection of the PP/PEI nanoparticles:plasmid DNA to adult wild-type mice, the mice were challenged with LPS and lung tissues were collected at 72 h post-LPS. As shown in Figure 4G, addition of the 3-kb *FOXM1* cDNA increasing the size of the plasmid from 12 kb to 15 kb did not affect the indel efficiency. Nanoparticle delivery of *FOXM1* cDNA resulted in increased FOXM1 expression (Figure 4H) and rescued the defective vascular repair (Figures 4I and 4J) in mice transduced with the CRISPR^*CDH5*^-*FOXM1* plasmid DNA.

### Highly efficient genome editing in ECs of the systemic vasculatures including heart, aortic, and peripheral vessels

**Figure 5.**
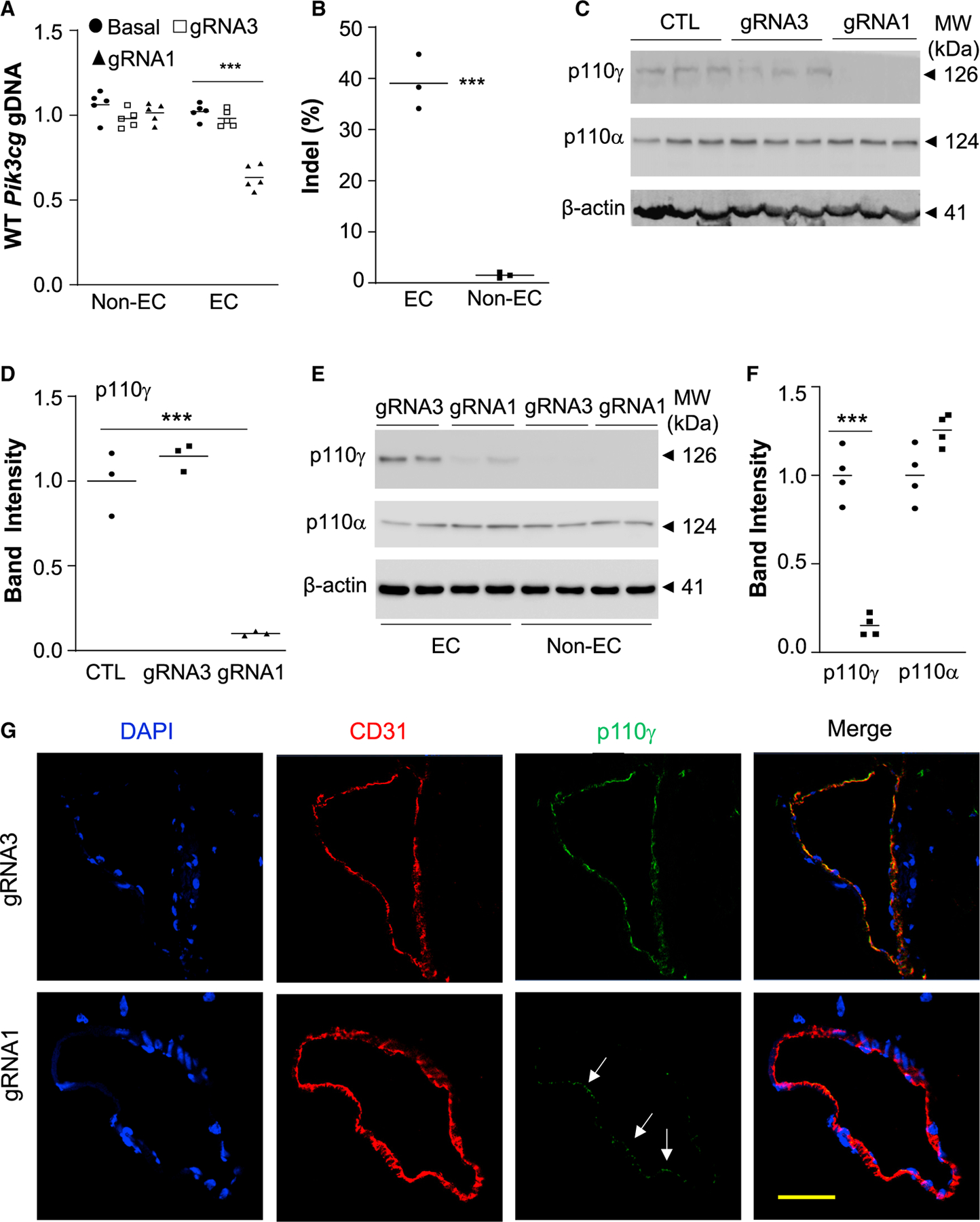
Highly efficient genome editing in cardiovascular ECs in adult mice (A) QPCR analysis demonstrating robust genome editing in cardiovascular ECs but not in non-ECs of *Pik3cg* gRNA1 nanoparticle-treated mice. At 7 days post-nanoparticle delivery, hearts were collected for EC isolation with anti-CD31 magnetic beads. ***p < 0.001, one-way ANOVA with Bonferroni post hoc analysis (n = 5). (B) Sanger sequencing decomposition analysis showing indels exclusively in cardiovascular ECs at a rate of 40% (n = 3). (C, D) Western blotting demonstrating greater than 80% decrease of p110γPI3K protein expression in hearts of CRISPR^*CDH5*^/gRNA1 nanoparticle-transduced mice compared with control (CTL) mice without plasmid DNA transduction. Quantification was carried out with Image J and normalized to loading control β-actin (n = 3). (E, F) Diminished expression of p110γPI3K in cardiovascular ECs of *Pik3cg* gRNA1 nanoparticle-treated mice (n = 4). (G) Representative immunofluorescent micrographs showing diminished p110γPI3K expression in cardiovascular ECs of gRNA1 nanoparticle-treated mice. Cryosections of mouse hearts were immunostained with anti-p110γ and anti-CD31 antibodies. Arrows point to ECs with less efficient knockdown of p110γPI3K. Scale bar, 50 μm. ***p < 0.001, one-way ANOVA with Bonferroni post hoc analysis (A, D), Student’s t test (B, F).

We then determined the genome editing efficiency in the systemic vasculature following PP/PEI nanoparticle delivery of CRISPR^*CDH5*^ plasmid DNA. QPCR analysis revealed an ~40% decrease in wild-type *Pik3cg* genomic DNA selectively in ECs obtained from hearts of gRNA1 but not gRNA3 plasmid-transduced mice (Figure 5A). Sanger sequencing decomposition analysis also revealed a 40% indel rate in ECs isolated from gRNA1-transduced mouse hearts (Figure 5B). Again, there was no genome editing in non-ECs. Western blotting demonstrated more than 85% decrease of p110γPI3K expression in hearts and ECs isolated from hearts of gRNA1 plasmid-transduced mice compared with those of gRNA3 plasmid-transduced mice (Figures 5C–5F). Immunofluorescent staining also showed a marked decrease of p110γPI3K expression in vascular ECs in hearts of gRNA1 plasmid-transduced mice (Figure 5G).

**Figure 6.**
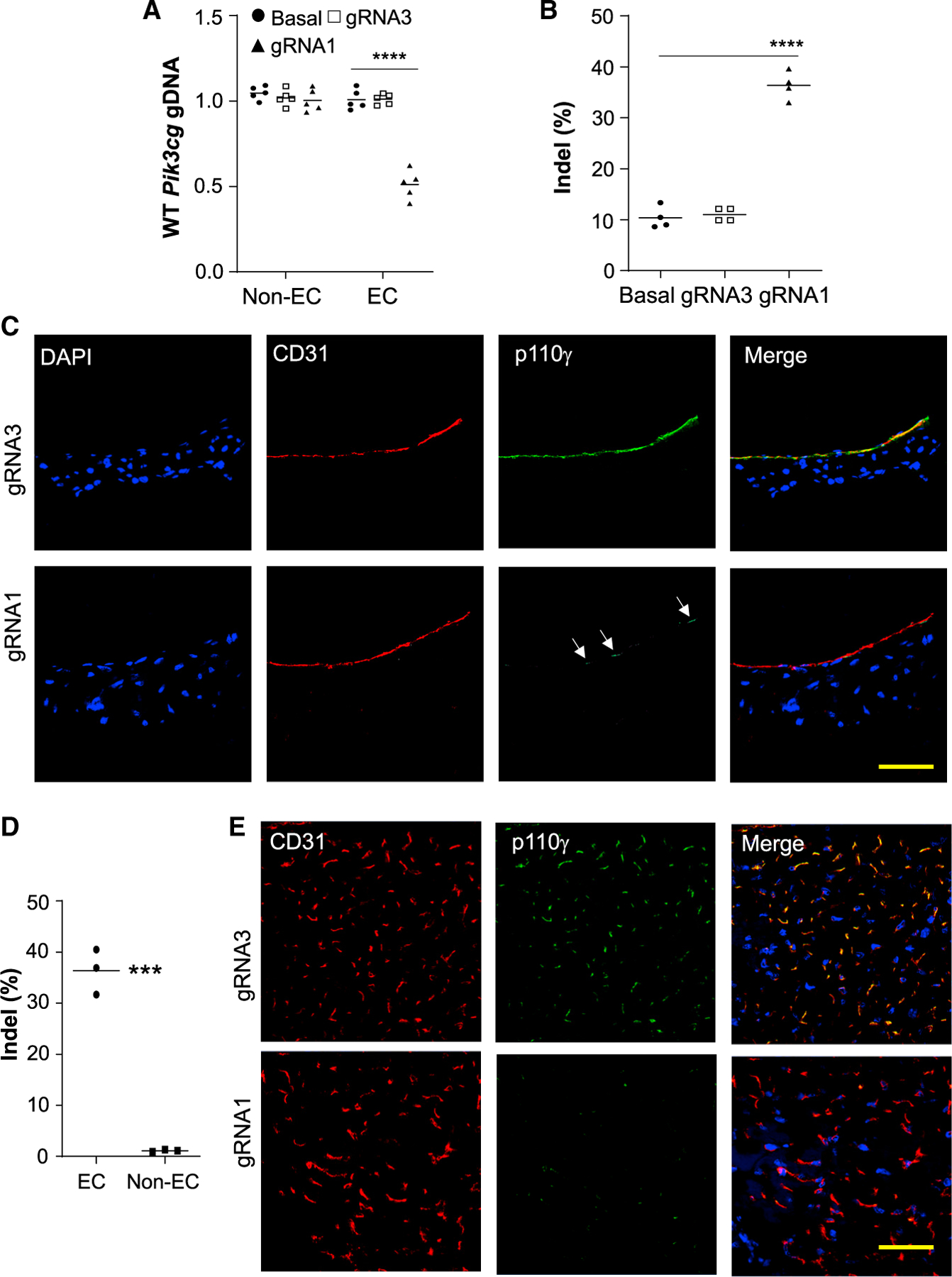
Robust genome editing selectively in vascular ECs of abdominal aorta and peripheral microvessels At 7 days post-nanoparticle delivery of CRISPR^*CDH5*^ plasmid DNA to adult mice, abdominal aorta and hindlimb skeletal muscle were collected and ECs were isolated with anti-CD31 magnetic beads. (A) QPCR analysis demonstrating efficient genome editing with selectivity for ECs of the abdominal aorta isolated from gRNA1 nanoparticle-treated mice (n = 5). (B) Sanger sequencing decomposition analysis showing greater than 40% indels in aortic ECs (n = 4). (C) Immunofluorescent staining showing that p110γPI3K expression was markedly decreased in aortic ECs in gRNA1 nanoparticle-treated mice in contrast to gRNA3-treated mice. Arrows point to ECs with less efficient knockdown of p110γPI3K. Scale bar, 50 μm. (D) Sanger sequencing decomposition analysis showing ~40% indels in skeletal muscle microvascular ECs but not in non-ECs in gRNA1 nanoparticle-treated mice (n = 3). (E) Immunofluorescent staining showing that diminished p110γPI3K expression in skeletal muscle microvascular ECs in gRNA1 nanoparticle-treated mice in contrast to gRNA3-treated mice. Scale bar, 50 μm. ***p < 0.001; ****p < 0.001. One-way ANOVA with Bonferroni post hoc analysis (A), Student’s t test (B, D).

In the abdominal aorta, we also detected an approximately 45% genome-editing efficiency in ECs isolated from the aorta of gRNA1 plasmid-transduced mice (Figures 6A and 6B). Genome editing was selectively induced in ECs. Immunofluorescent staining revealed diminished p110γPI3K expression in abdominal aortic vascular ECs of gRNA1 plasmid-transduced mice (Figure 6C). Together, these data demonstrate robust genome editing in ECs of the cardiovascular system by PP/PEI nanoparticle delivery of CRISPR^*CDH5*^ plasmid DNA in adult mice.

To assess the genome editing efficiency in the peripheral vasculature, we also isolated ECs and non-ECs from hindlimb skeletal muscle. Sanger sequencing decomposition analysis revealed a 37% indel rate in ECs but not in non-ECs (Figure 6D). Immunofluorescent staining also demonstrated diminished expression of p110γPI3K in skeletal muscle microvascular ECs of gRNA1 plasmid-transduced mice (Figure 6E). Intriguingly, we observed only 15% indel efficiency in cerebrovascular ECs (Figure S5D). There was no genome editing in bone marrow cells (Figure S5E) while marginal indels in hepatocytes (Figure S5F) of the CRISPR^*CDH5*^ plasmid DNA-transduced mice. Furthermore, when we delivered the CRISPR^*CAG*^ plasmid to adult mice, there was no genome editing in bone marrow cells, whereas 20% indels in hepatocytes. These data demonstrate our system is more efficient in targeting the cardio and pulmonary vasculatures than hepatocytes and bone marrow cells *in vivo*.

### Simultaneous genome editing of two genes by one CRISPR^CDH5^ plasmid DNA

We next addressed the possibility of genome editing of two genes simultaneously by nanoparticle delivery of a CRISPR plasmid DNA expressing two gRNAs (Figure S6A). Adult mice were administered retro-orbitally with the mixture of PP/PEI nanoparticles: *CRISPR*^*CDH5*^ plasmid DNA expressing either *Pik3cg* gRNA1, *Vegfr2* gRNA3 or both. The plasmid DNA expressing two gRNAs resulted in genome editing of both genes at high efficiency similar to the single gRNA plasmid (Figure S6B).

### Endothelium-targeted genome editing of Vegfr2 in adult mice is sufficient to induce spontaneous disease development

**Figure 7.**
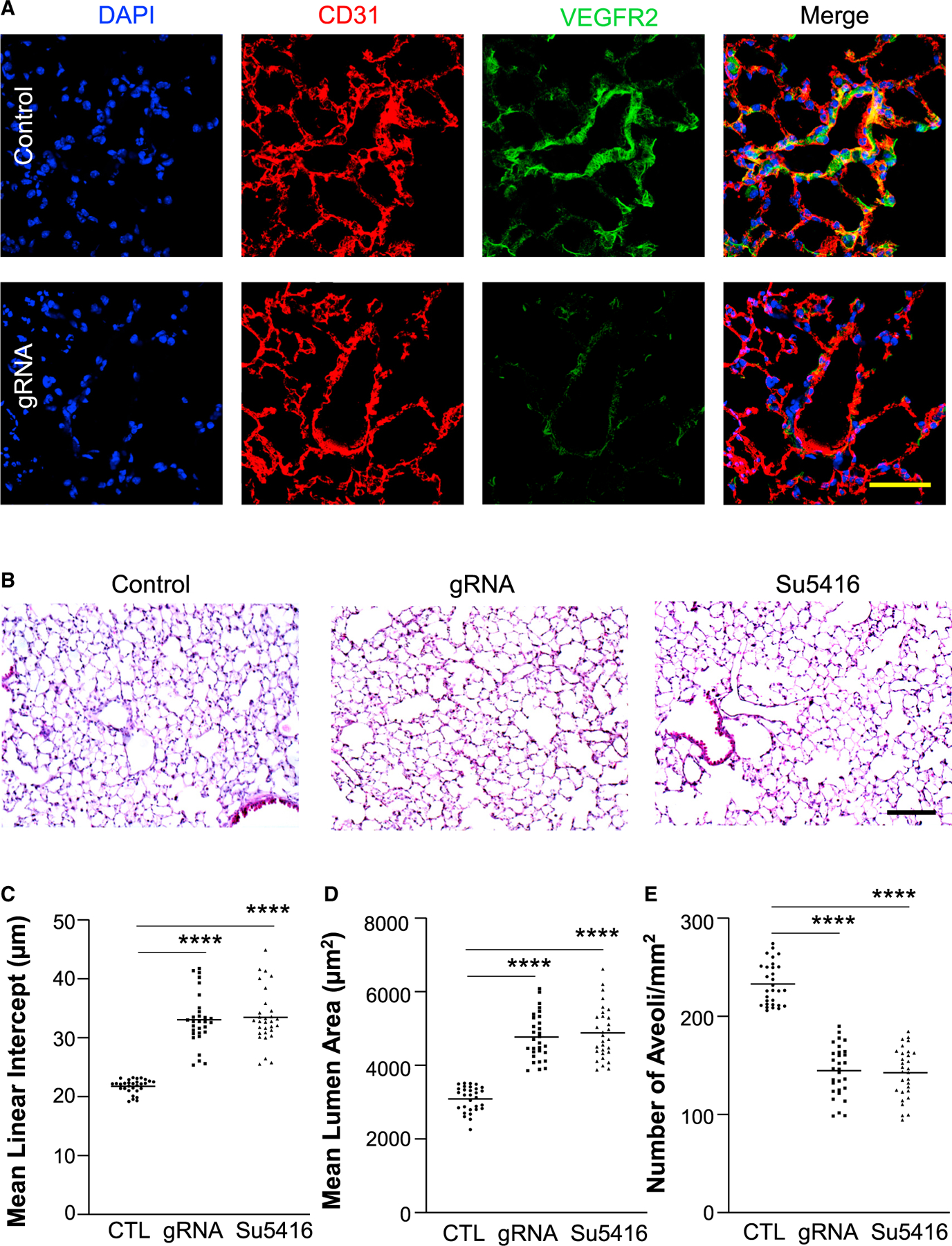
Genome editing-mediated disruption of Vegfr2 in ECs causes emphysema-like phenotype in adult mice similar to Sugen5416 inhibition of VEGFR-induced changes (A) Representative micrographs of immunostaining demonstrated diminished VEGFR2 expression in lung vascular ECs of CRISPR^*CDH5*^/*Vegfr2* gRNA plasmid-transduced mice. Anti-CD31 was employed to immunostain ECs (red). Control = CRISPR^*CDH5*^ plasmid with scrambled RNA. V, vessel. Scale bar, 50 μm. (B) Representative micrographs of H&E staining of lung sections. Control = CRISPR^*CDH5*^ plasmid without gRNA. Complexes of PP/PEI nanoparticle:plasmid DNA expressing Vegfr2 gRNA (gRNA) or scramble (Control) were administered to 3-month-old WT mice at day 1 and day 8 and lung tissues were collected at day 28. A separate cohort of mice was treated with Sugen5416 three times a week for 21 days and lung tissues were collected at day 28. Scale bar, 200 μm. (C–E) Quantification of mean linear intercept (C), mean lumen area (D), and number of alveoli/mm^2^ (E). n = 5 mice/group and 6 fields/mouse. ****p < 0.0001. One-way ANOVA with Dunnett’s multiple comparisons test.

Immunostaining revealed diminished Vegfr2 expression in pulmonary vascular ECs of *Vegfr2* gRNA plasmid DNA-transduced mice (Figure 7A). Similarly, Vegfr2 expression was also markedly disrupted in heart vascular ECs (Figure S7A) and aortic vascular ECs (Figure S7B). It has been shown that inhibition of vascular endothelial growth factor (VEGF) signaling by genetic disruption of *Vegf* (Tang et al., 2004), pharmacological blockade of VEGFR signaling (Kasahara et al., 2000), or small interfering RNA (siRNA)-mediated knockdown of *Vegfr2* in ECs (Dahlman et al., 2014) induces emphysema, a disease characterized by decreased pulmonary surface area. We next determined if nanoparticle delivery of *Vegfr2* gRNA plasmid DNA-mediated knockout of *Vegfr2* in ECs also induced emphysema. As a positive control, a separate cohort of wild-type mice were treated with the VEGFR inhibitor Sugen 5416 (Kasahara et al., 2000). Four weeks after the nanoparticle:*Vegfr2* gRNA plasmid DNA administration or Sugen 5416 treatment, lung tissues were collected for histological assessment. As shown in Figures 7B–7E, *Vegfr2* gRNA-mediated knockout of *Vegfr2* induced emphysema evident by marked increases of mean linear intercept and mean lumen area of alveolus as well as reduced number of alveoli, which were similar to those of Sugen 5416-treated mice. These data demonstrate that PP/PEI nanoparticle delivery of CRISPR-Cas9 system leads to robust genome editing and thus gene disruption in ECs in adult mice, which is sufficient to drive a phenotype similar to pharmacological blockade of the signaling or genetic knockout mice.

## DISCUSSION

In the present study, we report a simple and highly efficient approach to induce genome editing selectively in the vascular endothelium through non-viral delivery of CRISPR plasmid DNA with the aid of a PEG-*b*-PLGA copolymer-based nanoparticle. We showed that PP/PEI nanoparticle, coupled with human *CDH5* promoter-driven expression of Cas9, induces robust EC-restricted genome editing within 7 days in multiple organs, including heart, lung, aorta, and peripheral vessels of adult mice by a single i.v. administration, and thereby causes diminished protein expression in ECs, which results in a phenotype similar to that seen in genetic knockout mice. The genome editing efficiencies in wild-type mice were similar under normal and pathological conditions. Using one plasmid DNA, we also achieved genome editing of two genes simultaneously as well as genome editing of one gene and expression of a transgene for functional rescue.

AAV has thus far been considered to be the most suitable vehicle for delivering genome editing system locally or systemically (Cox et al., 2015; Schmidt and Grimm, 2015; Varshney et al., 2015). The immunogenicity of AAV and its packaging size limitation restrict the applicability of AAV (Mingozzi and High, 2013; Wu et al., 2010). Another concern is that extended expression of Cas9 may cause unwanted DNA damage. Thus, recent studies focused on development of non-viral delivery of CRISPR system components. Engineered Cas9 ribonucleoprotein complexes that were locally delivered to cells *in vivo* by cationic liposomes or lipofectamine 2000 caused genome editing but with limited efficiency (Yuen et al., 2017; Zuris et al., 2015). Recent studies show that lipid nanoparticles coupled with chemically modified gRNAs have induced efficient genome editing in mouse livers (Finn et al., 2018; Yin et al., 2016, 2017); however, selective and highly efficient targeting of specific organs and cell types other than the liver and hepatocytes remains challenging (Cox et al., 2015; Varshney et al., 2015). Luo et al. show that cationic lipid-modified PEG-*b*-PLGA nanoparticles by encapsulating plasmid DNA expressing Cas9 (under the control of the *CD68* promoter) and gRNA induce macrophage-specific genome editing with 20% indel rate and 30% to 40% reduction of protein levels (Luo et al., 2018). Our study has demonstrated robust EC-restricted *in vivo* genome editing in various vascular beds with 40% to 50% indel rate and ~80% reduction of protein levels. Although both nanoparticles employ PEG-*b*-PLGA, Luo et al. (2018) modify the polymer nanoparticle with cationic lipid to promote cell uptake and the plasmid DNA is directly encapsulated inside the nanoparticles while we conjugate the PEG-*b*-PLGA nanoparticles with PEI on the surface, which further forms complex with plasmid DNA. Our studies have demonstrated the highest efficiency of disruption of protein expression *in vivo*.

PEGylation of nanoparticles has been shown to increase systemic circulation time by inhibiting nanoparticle aggregation, opsonization, and phagocytosis (Suk et al., 2016). Sustained nanoparticle circulation will make it possible to deliver the payload to various tissues. PEGylation also results in different tissue distribution of the nanoparticles. It is shown that PEGylated gold nanorods accumulate the most in the spleen, whereas the non-PEGylated gold nanorods accumulate in the liver (Lankveld et al., 2011). Our data show that nanoparticles made with PEG-*b*-PLGA with large molecular weight PEG_5000Da_ have better tissue distribution than either PLGA nanoparticles or PEG_600Da_-PLGA nanoparticles, which is essential for targeting of the vascular system. Notably, we employed a one-step approach with PEG-*b*-PLGA as the starting material instead of the two-step approach with PLGA as the starting material to generate PLGA nanoparticles followed by PEGylation. Our study has identified that PEG_5000_-*b*-PLGA nanoparticles have excellent biodistribution after systemic administration and induce highly efficient *in vivo* genome editing of various vascular beds, including heart, lung, and aorta as well as the peripheral vasculature. We observed highly efficient genome editing in not only large vessels such as the abdominal aortic vessels but also the small/micro vessels such as the skeletal muscle microvessels.

Our biodistribution data show that PP/PEI nanoparticles and PP/PEI/plasmid DNA were accumulated in liver and lung comparably. However, there was minimal genome editing in hepatocytes with the CRISPR^*CDH5*^ plasmid, which is ascribed to the specificity of the *CDH5* promoter. Using the universal *CAG* promoter, we observed only 20% genome editing in hepatocytes in the liver and no genome editing in bone marrow cells. These data demonstrate EC-enriched uptake of the nanoparticles *in vivo*. Following i.v. administration, the nanoparticles are first taken up by vascular ECs. It is possible that the nanoparticles are not efficient in passing through the endothelial barrier for uptake by tissue resident cells, such as hepatocytes in liver. In cultured hepa-1c1c7 (a hepatoma cancer cell line), the nanoparticle could be efficiently uptaken and induced highly efficient genome editing (e.g., *Pik3cg* gRNA1 causes >80% indel efficiency). Thus, it seems the bioavailability of the nanoparticles determines the selectivity of vascular ECs. Coupled with the EC-specific promoter to control Cas9 expression, our system can exclusively target ECs in all cardio and pulmonary vasculatures with a pan-endothelial promoter (e.g., *CDH5*) or a specific vasculature with an organ-specific endothelial promoter.

Besides tissue- and cell-specific targeting, there are several advantages of non-viral delivery of the CRISPR-Cas9 genome-editing system, as used here. First, there is greatly reduced risk of long-term Cas9 expression-mediated adverse effects. Our data show that CRISPR plasmid DNA delivered by nanoparticles was almost fully cleared in various organs including spleen, kidney, liver, thymus, lung, aorta, and heart after 6 days of administration. Second, the plasmid DNA used is not only easy and inexpensive to prepare but also more stable than RNA or protein. Third, there is unrestricted packing size. Our study demonstrated that the size of the plasmid at 15 kb did not affect the efficiency of genome editing. We used the same CRISPR plasmid to introduce genome editing of one gene and expression of a 3-kb transgene in the same cells. We have also shown similar efficiency in inducing genome editing of two genes simultaneously with one plasmid. Future study will explore if one plasmid DNA can induce robust genome editing of three or even more genes. Thus, this system can induce robust genome editing of at least two genes and also introduce transgene expression in the same cells. Such a strategy should greatly facilitate the delineation of gene functions in the vascular system with potential for non-viral gene therapy of vascular diseases.

In addition to Cas9 and Cas9-based systems, our strategy may also be employed to deliver other CRISPR systems such as Cas13, which induces RNA knockdown (Abudayyeh et al., 2017), and Cpf1 (Zetsche et al., 2015), base editors, or the prime editing system to target the endothelium. The successful use of the human *CDH5* promoter here in mice suggests that our system should be effective in targeting the human endothelium. Thus, with the development of more specific CRISPR systems with reduced off-target effects (Chen et al., 2017; Kleinstiver et al., 2016; Slaymaker et al., 2016) as well as base editors and the prime editing system, this strategy may have great potential for therapeutic genome editing in humans to prevent and treat vascular diseases.

There is another aspect of important technique we developed here. To quantify indels, next generation sequencing is a gold standard. Sanger sequencing decomposition analysis using TIDE software (Brinkman et al., 2014; Yin et al., 2017) is a relatively simple method. However, both methods are quite labor-intensive. We developed a quantitative PCR method using a primer targeting the predicted cleavage site to quantify genome editing. The primer cannot amplify the mutated DNA with deletions and insertions near the predicted cleavage site due to 3′ mismatch. The indel rate revealed by QPCR is comparable to the data from next generation sequencing analysis. Our validation study has demonstrated that QPCR analysis can efficiently distinguish wild-type DNA from mutant DNA with 1-base pair deletion or insertion. Thus, the QPCR-based genomic DNA analysis is a simple and reliable method for indel quantification.

In summary, we developed a PEG-*b*-PLGA copolymer-based nanoparticle system to deliver the CRISPR-Cas9 plasmid DNA to the vasculature of multiple organs and induce highly efficient genome editing in vascular ECs following a single i.v. administration that causes diminished protein expression selectively in ECs to generate a phenotype similar to that seen in genetic knockout mice. It can induce genome editing of multiple genes and also introduce transgene expression simultaneously. This simple and efficient approach for EC-specific *in vivo* genome editing as well as transgene expression in adults will provide a powerful research tool to quickly delineate gene function in the cardiopulmonary vascular system to facilitate our understanding of molecular mechanisms and identify druggable targets for vascular diseases. The similar genome editing efficiency under normal and pathological conditions indicates the potential of future development of this technology for therapy of vascular diseases by genome editing and gene transfer.

### Limitations of the study

There are some limitations to our study. First, the mechanism(s) underlying the EC-enriched uptake of PP/PEI nanoparticles remains elusive. The PP/PEI nanoparticles can also be uptaken by hepatocytes and induce genome editing in hepatocytes, although much less efficiently. To achieve EC-specific genome editing, the study also employs the EC-specific promoter to control Cas9 expression selectively in ECs. Second, the genome editing efficiency in brain vascular ECs is only modest. It is unknown whether brain vascular ECs are less efficient in uptaking the nanoparticles, and/or the *CDH5* promoter is not as strong as in other vascular beds since the brain vascular ECs are quite different from cardiopulmonary vascular ECs. It is interesting to test a stronger promoter of a brain vascular EC gene in a future study. Third, the mechanism(s) of disproportionately large benefits of protein knockdown of modest genome editing is unclear. Although we observed only 30% to 50% genome editing in vascular ECs *in vivo*, which is highly efficient compared with the data in the literature, protein levels were decreased ~80% and the mice exhibited phenotypes similar to those seen in genetic knockout mice or mice with a pharmacological inhibitor. This disproportional effect of changes in genomic DNA levels on protein level changes is also well-known in the literature (Koblan et al., 2021). Future study is warranted to delineate the underlying mechanisms.

## STAR★METHODS

### RESOURCE AVAILABILITY

#### Lead contact

Further information and requests for resources and reagents should eb directed to and will be fulfilled by the Lead Contact, You-Yang Zhao (youyang.zhao@northwestern.edu).

#### Materials availability

The Plasmids in this study were generated from the materials available in Addgene. Please contact the Lead Contact for further information. The nanoparticles are available from Mountview Therapeutics LLC or the Lead Contact.

#### Data and code availability

- This study didn’t generate any unique dataset that is central to supporting the main claims of the paper.
- This study didn’t generate any code.
- Any additional information required to reanalyze the data reported in this work paper is available from the Lead Contact upon reasonable request.

### EXPERIMENTAL MODEL AND SUBJECT DETAILS

#### Mice

C57BL/6J mice (male and female) at 3–5 months of age (originally from The Jackson Laboratory) were used for *in vivo* genome editing. All mice were bred and maintained in the Association for Assessment and Accreditation of Laboratory Animal Care-accredited animal facilities at Northwestern University according to National Institutes of Health guidelines. All animal experiments were performed in accordance with protocols approved by Northwestern University Institutional Animal Care and Use Committee.

#### Mouse Hepa-1c1c7 cell line

Hepa-1c1c7 cells were purchased from the American Type Culture Collection (ATCC). Cells were maintained in DMEM supplemented with 10% FBS and penicillin/streptomycin. Cells were split and grown to 40–50% prior to transfection.

### METHOD DETAILS

#### Preparation of CRISPR plasmids

Preparation of the all-in-one CRISPR-Cas9/gRNA plasmid DNA was performed as described previously (Ran et al., 2013). Briefly, the single complementary DNA oligonucleotides corresponding to the gRNA sequence were commercially synthesized (Integrated DNA Technologies). After phosphorylation and annealing, the paired double strand DNA oligo was cloned into the BbsI linearized plasmid p*Sp*Cas9(BB)-2A-GFP. Positive clones containing the gRNA-encoded DNA sequences were confirmed by DNA sequencing. For EC-specific genomic editing, the *CAG* promoter in p*Sp*Cas9(BB)-2A-GFP plasmid (CRISPR^*CAG*^) was replaced with the human *CDH5* promoter (Gory et al., 1999) using Kpn I and Age I restriction enzyme sites (CRISPR^*CDH5*^). All gRNA sequences are listed in Table S1.

To generate CRISPR^*CDH5*^ plasmid expressing both *Pik3cg* gRNA and FOXM1, the P2A and FOXM1 cDNA fragment was inserted into BsrGI linearized CRISPR^*CDH5*^ plasmid DNA expressing *Pik3cg* gRNA using NEBuilder HiFi DNA assembly (New England Biolabs).

Dual targeting CRISPR plasmid DNA expressing *Pik3cg* gRNA and *Vegfr2* gRNA was generated by inserting cassette of U6-Vegfr2 gRNA into CRISPR^*CDH5*^ plasmid DNA expressing *Pik3cg* gRNA using Xba I and Kpn I sites. All plasmids were confirmed by DNA sequencing.

#### Preparation of PP/PEI nanoparticles

PP/PEI nanoparticles were prepared by two steps (Mountview Therapeutics LLC). First, PP nanoparticles were synthesized by emulsification and evaporation. Briefly, PEG-*b*-PLGA copolymer (Polysci Tech, Akina) were dissolved in dichloromethane and homogenized to form the oil phase emulsification. The oil phase emulsification was combined and homogenized to form the second water phase emulsification. The PP nanoparticles were harvested by centrifugation. Second, the synthesized PP nanoparticles were mixed with PEI_25,000_ (Sigma Aldrich) and incubated at room temperature for 72 h. The size of the harvested PP/PEI nanoparticles was estimated by dynamic laser scattering using a Zetasizer Nano ZS (Malvern Instruments, UK).

#### *In vitro* identification of potent gRNA using PP/PEI nanoparticle:CRISPR-Cas9 plasmid complex

Hepa-1c1c7 cells (ATCC) were maintained in DMEM with 10% FBS, 100U/ml penicillin, and 100μg/ml streptomycin. The all-in-one CRISPR^*CAG*^ plasmid DNA was transfected to Hepa-1c1c7 cells (cell density 50–70%) in complete medium, i.e., without starvation, using PP/PEI nanoparticles. At 72 h post-transfection, the transfected cells were collected for genomic DNA (gDNA) isolation. The gDNA was used for quantitative real-time PCR analysis to identify the highly potent gRNA(s). The gDNA containing the gRNA-target sequence was also amplified by PCR and the PCR product was then used for Sanger DNA sequencing to determine indels using Tide software analysis (Brinkman et al., 2014).

#### Determination of living tissue distribution of PP and PP/PEI nanoparticles using fluorescence tomography

Various nanoparticles comprised of PLGA polymer or PEG-b-PLGA copolymer or PP/PEI used for living tissue distribution were prepared as the normal procedure described above except the oil phase containing 100 μg of coumarin 6 (Sigma Aldrich) as the fluorescence indicator. At 8 h after administration of nanoparticles by retro-orbital injection, various organ tissues were collected for determination of tissue distribution using IVIS Lumina II preclinical imaging system (PerkinElmer). To quantify the fluorescent intensity of coumarin6, the tissues were homogenized in 5% DMSO for 5 min, and centrifuged for 10 min at 21,000*g*. The emission of coumarin6 in supernatant was measured at 534 nm after excitation at 444 nm. The amount of coumarin 6 was calculated using a coumarin standard curve and normalized to tissue weight (ng/mg tissue).

Fluorescence tomography was also employed to determine the tissue distribution of the nanoparticles in live mice at 5 h post-administration. The fluorescence was measured at 625 nm following 545nm excitation by the cold CCD camera. The illuminated and fluorescent images were analyzed using the IVIS living imaging software 4.4.

#### Nanoparticle delivery of CRISPR^*CDH5*^ plasmid DNA for *in vivo* genome editing in adult mice

The characterized nanoparticles were mixed with CRISPR^*CDH5*^ plasmid DNA following the manufacture’s instruction and kept at room temperature for 10 min before use. Each adult mouse was given 40 μg plasmid DNA via retro-orbital injection. 4–7 days after nanoparticle delivery, the mice were used for experiments.

#### CRISPR plasmid DNA biodistribution and pharmacokinetics

The CRISPR plasmid DNA was delivered to mice by nanoparticles through retro-orbital injection. The mice were then scarified at the indicated time, and various organs were collected after bloodletting through abdominal aorta. The tissues were weighed and digested by proteinase K overnight. The supernatants were collected after centrifugation at 20,000*g* for 10 min. The CRISPR plasmid DNA in supernatants was precipitated by equal volume of ethanol and used to quantify Cas9 DNA fragment by QPCR. The Cas9 DNA amount was calculated using standard curve generated from the CRISPR plasmid DNA and normalized according to the tissue weight (ng DNA/g tissue). The QPCR primers are as following, forward 5′-CATCGAGCAGATCAGCGAGT-3′ and reverse 5′-ATCCCGGTGCTTGTTGTAGG-3′.

#### Next generation sequencing

Two-step PCR was carried out to generate library for next generation sequencing. Briefly, first PCR amplified the fragments located in the predicted editing region. Second PCR barcoded the fragments. All fragments were then purified by agarose gel electrophoresis. The sequencing was performed using Illumina standard sequencing platform. The original FASTQs were obtained after demultiplexing. The FASTQs were aligned to amplicon reference and quantified using CRISPResso2 (http://crispresso.pinellolab.partners.org/). The forward primer is 5′-ACACTCTTTCCCT.

ACACGACGCTCTTCCGATCTTGTGCTCTTCCTTTAGGCTGT-3′, and the reverse primer is 5′-GTGACTGGAGTTCAGACGTGTGCTCTTCCGATCTGCTTCAGCAGG.

AATCTGGC-3’. The underlined sequences are primer sequences for genomic DNA amplification. The entire assembly fragment for sequencing is 207 nucleotides and the sequence product of *Pik3cg* genomic region is 71 nucleotides.

#### Quantitative real-time PCR analysis of genome editing

Genomic DNA was extracted. SYBR Green-based quantitative real-time PCR analysis (Roche Applied Science) were performed with the 7500 fast Real-Time PCR System (Thermo Fisher Scientific). The forward primer was designed to end with the predicted cleavage site and thus it will not amplify mutant DNA with unmatched 3’. The primer sequences for analysis of *Pik3cg* gRNA1-mediated genome editing and Vegfr2 gRNA3-mediated genome editing were listed in Table S2.

To determine if QPCR can distinguish DNA with single bp deletion or insertion from wild-type DNA, mutant CRISPR^*CDH5*^ plasmid DNA (CRISPR^Del^ or CRISPR^Ins^ plasmids) were made by replacing the wild-type Cas9 fragment with a mutant Cas9 fragment containing single base deletion or insertion using BglII and EcoRV sites. A forward primer matching the wild-type sequence was used to amplify wild-type fragment versus mutant fragment with 1 bp deletion. A forward primer with the 3′insertion nucleotide was used to amplify the mutant fragment with 1bp insertion versus wild-type fragment.

#### LPS-induced endotoxemia in mice

LPS (E. coli 055:B5, Santa Cruz) was administered i.p. to mice at a dose of 2.5 mg/kg body weight in PBS (6 μl/g). 72 h after LPS administration, the mice were euthanized for tissue collection.

#### Isolation of ECs and non-ECs from mouse tissues

After perfused free of blood with PBS, lung, heart, abdominal aorta, hindlimb skeletal muscle, or brain was cut into small pieces, and then incubated with 1 mg/ml collagenase A (Roche Applied Science) for 1 h at 37°C in a shaking water bath (200rpm). After digestion, the tissue was dispersed to a single cell preparation using the gentleMACS™ Dissociator (Miltenyi Biotec) with lung program 2 (which also works well with heart, aorta, skeletal muscle, and brain). The cells were then filtered using a 40 μm Nylon cell strainer and blocked with 20% FBS for 30 min. After 15 min incubation with Fc blocker (1 μg/10^6^ cells, BD Biosciences), the cells were incubated with anti-CD31 (1:1000, BD Biosciences) for 30 min at room temperature. After washing twice, the immunostained cells in 1 ml PBS were added with 50 μl pre-washed Dynabeads conjugated with anti-rat IgG secondary antibody, and incubated for 30 min at room temperature. The cells were then subjected to magnetic purification (Dai et al., 2018). After washing twice, the cells were used for experiments. Flow cytometry analysis demonstrated that the purity of ECs isolated by magnetic sorting was ~90%. Non-ECs were collected from the wash-through cells after 2 times anti-CD31 incubation and depletion.

#### Isolation of hepatocytes

Hepatocyte isolation was performed according to previously described protocol with slight modification (Charni-Natan and Goldstein, 2020). Mice were perfused for whole body using HBSS without Ca^2+^, Mg^2+^, and phenol red until liver became gray. The liver tissues were cut into small pieces, and washed twice using HBSS, and then digested for 1 h using 25μg/ml Liberase (3 ml per 100 mg tissues) in 37°C water bath with 200rpm shaking. After digestion, liver tissues were dispersed using liver program with the gentleMACS™ Dissociator and then filtered using a 40 μm cell strainer. The hepatocytes were collected by centrifugation, and incubated for 5 min in red cell lysis buffer. After centrifuge, the hepatocytes were resuspended in 5 ml DMEM, and added 5 ml freshly made Percoll solution. The hepatocytes and Percoll solution were mixed thoroughly by inverting tube several times. The hepatocytes located in bottom after centrifuge at 200*g* for 10min were collected for genomic DNA extraction.

#### Lung transvascular albumin flux assessment

The EBA flux assay was carried out as described previously (Huang et al., 2016). Briefly, Evans blue dye (Sigma Aldrich) was dissolved in PBS at 15 mg/ml with slow shaking at room temperature for 3 h and the solution was collected after centrifugation. Bovine serum albumin (fraction V, Sigma Aldrich) was also dissolved in PBS (8 mg/ml) and purified with charcoal (Sigma Aldrich) by mixing 150 mg of albumin with 300 mg of charcoal in 12.5 ml PBS. Following vortexing (30 sec, 10 times), the solution was incubated for 1 h at room temperature with slow shaking and then centrifuged at full speed (13,000rpm) for 5 min. The supernatant was collected and centrifuged for another 5–6 times until there were no particles in the supernatant. Evans blue and albumin solutions were mixed at a 1:2 ratio and incubated for 45 min with slow shaking at room temperature and then sterile-filtered through a 0.22 μm syringe filter. EBA (20 mg/kg BW) was retro-orbitally injected into mice 40 min before tissue collection. Lungs were perfused free of blood with PBS, blotted dry, weighed and snap frozen in liquid nitrogen. The right lung was homogenized in 0.5 ml PBS and incubated with 1ml formamide at 60°C for 18h. The homogenate was then centrifuged at 21,000 × g for 10 min and the optical density of the supernatant was determined at 620 nm and 740 nm. Extravasated EBA in lung homogenates was expressed as micrograms of Evans blue dye per g lung tissue.

#### Myeloperoxidase assay

Lung tissues perfused free of blood with PBS were homogenized in 5 mM (0.5 ml) phosphate buffer (pH 6.0) and then centrifuged at 21,000 ×g for 10 min at 4°C (Huang et al., 2016). The pellets were resuspended in phosphate buffer containing 0.5% hexadecyl trimethylammonium bromide (Sigma Aldrich) and subjected to a cycle of freezing and thawing. Subsequently the pellets were homogenized and the homogenates were centrifuged again. The supernatants were assayed for MPO activity (Huang et al., 2016) by mixing 50 μl of sample, 75 μl of 0.015% H_2_O_2_, and 15 μl of O-dianisidine dihydrochoride solution (16.7mg/ml) in 1.38 ml of phosphate buffer, and reading absorbance at 460 nm every 20 sec for 3 minutes. Results are expressed as ∆OD_460_/min/g lung tissue.

#### Induction of emphysema and characterization

Sugen 5416 (Cayman Chemicals) (20 mg/kg), dissolved in PBS containing 20% DMSO, was injected intraperitoneally into wild-type mice (4 weeks of age) three times a week for 21 days. Separate cohorts of mice were injected retro-orbitally with mixture of CRISPR^*CDH5*^ plasmid expressing *Vegfr2* gRNA and PP/PEI nanoparticles at days 1 and 8. The lung tissues were collected and fixed at day 28. 5 μm paraffin sections were stained with hematoxylin and eosin. The images were taken using light microscopy. The mean linear intercept was quantified using ImageJ with MLI plugin (Crowley et al., 2019). The mean lumen area and alveoli number were determined using ImageJ with angiogenesis analyzer plugin (Carpentier et al., 2020).

#### Confocal microscopy

Cryosections (3–5 μm) of mouse tissues (perfused free of blood with PBS) were fixed with 4% paraformaldehyde and then immunostained with anti-p110γ antibody (Cat#5405, 1:200, Cell Signaling Technology). The section was also immunostained with anti-CD31 (Cat# 557355, 1:100, BD Biosciences) to identify vascular ECs. Nuclei were counterstained with DAPI (Prolong Gold Antifade Mountant with DAPI, Thermo Fisher Scientific). Sections were imaged with a confocal microscope system (LSM 880; Carl Zeiss, Inc) equipped with a 40 × 1.30 NA oil DIC M27 objective lens (Carl Zeiss, Inc.).

#### Molecular analysis

Mouse tissues were lysed in Trizol reagent (Thermo Fisher Scientific) using the TissueLyser (Qiagen) and total RNA was purified using the RNeasy mini kit including DNase I digestion (Qiagen) according to manufacturer’s instructions. Total RNA from cultured cells was isolated directly using the RNeasy mini kit. Following conversion of RNA to cDNA with reverse transcriptase (Applied Biosystems), SYBR Green-based quantitative real-time PCR analysis (Roche Applied Science) was performed with the 7500 fast Real-Time PCR System (Thermo Fisher Scientific). All quantitative PCR primers are listed in Table S3.

Tissue and cell lysates in RIPA buffer were used for Western blotting with the following antibodies: anti-p110γ (Cat#5405, 1:1000, Cell Signaling Technology), anti-p110α (Cat#4255, 1:1000, Cell Signaling Technology), and anti-β-actin (Cat#612656, 1:3000, BD Biosciences).

### QUANTIFICATION AND STATISTICAL ANALYSIS

Statistical differences between multiple groups were determined by one- or two-way *ANOVA* with Bonferroni or Dunnett post-hoc multiple analysis. Two-group comparisons were analyzed by the two-tailed unpaired Student’s *t* test. *P* < 0.05 denoted the presence of a statistically significant difference.

## Supplementary Material

1

## Figures and Tables

**Table:** KEY RESOURCES TABLE

REAGENT or RESOURCE	SOURCE	IDENTIFIER
Antibodies		

p110αPI3K	Cell Signaling Technol	Cat# 4255; RRID: AB_65988
p110γPI3K	Cell Signaling Technol	Cat# 5405; RRID: AB_1904087
CD31	BD Biosciences	Cat# 557355; RRID: AB_396660
VEGFR2	Cell Signaling Technol	Cat# 9698; RRID: AB_11178792
β-actin	BD Biosciences	Cat# 612656; RRID: AB_2289199

Chemicals, peptides, and recombinant proteins		

poly(lactide-co-glycolide) (PLGA, 55kDa)	PolySci Tech, Akina	Cat# AP121
poly(ethylene glycol) methyl ether-*block*-poly(lactide-co-glycolide) (PLGA-PEG_600Da_)	Nanosoft Polymers	Cat#2753-55k-600 Custom
poly(ethylene glycol) methyl ether-*block*-poly(lactide-co-glycolide) (PLGA-PEG_5000Da_)	PolySci Tech, AKina	Cat# AK026
Polyethyleneimine (MW25000Da)	Millipore Sigma	Cat#: 408727
Sugen 5416	Cayman Chemical	Cat# 13342
Coumarin-6	Millipore Sigma	Cat# 442631

Experimental models: Cell lines		

Hepa-1c1c7	ATCC	Cat# CRL-2026

Experimental models: Organisms/strains		

Mouse: C57BL/6J	The Jackson Laboratories	Cat# 000664 In house breeding

Oligonucleotides		

See Table S1 for gRNA sequences targeting mouse *Pik3cg* and *Vegfr2*	Integrated DNA Technologies	Custom
See Table S2 for primer sequences For genome editing analysis	Integrated DNA Technologies	Custom
See Table S3 for primer sequences for gene expression analysis	Integrated DNA Technologies	Custom

Recombinant DNA		

All-in-one CRISPR^*CAG*^ plasmid DNA	(Ran et al., 2013)	RRID: Addgene_48138
*CDH5* promoter	(Prandini et al., 2005)	N/A
*FOXM1 cDNA*	(Huang et al., 2016)	Gene bank: U74613.1
All-in-one CRISPR^*CDH5*^ plasmid DNA expressing Cas9 and *Pik3cg* gRNA	This work	N/A
All-in-one CRISPR^*CDH5*^ plasmid DNA expressing Cas9 and *Vegfr2* gRNA	This work	N/A
All-in-one CRISPR^*CDH5*^ plasmid DNA expressing *Pik3cg* gRNA and *Vegfr2* gRNA for double knockout	This work	N/A
All-in-one CRISPR^*CDH5*^ plasmid DNA expressing *Pik3cg* gRNA and FOXM1 transgene for *Pik3cg* knockout and overexpression of FOXM1 simultaneously	This work	N/A

## Highlights

- PPP i.v. exhibits excellent biodistribution without specific liver accumulation
- PPP efficiently delivers plasmid DNA *in vivo* targeting vascular ECs
- PPP delivery of CRISPR plasmid decreases 80% protein in cardiopulmonary vascular ECs
- The system induces genome editing of two genes in ECs without limitation on plasmid size
