# Supplementary material for: Robust genome editing in adult vascular endothelium by nanoparticle delivery of CRISPR-Cas9 plasmid DNA
Source: Cell Rep. Author manuscript; Available in PMC 2022 Jan 19. (PMC8769807; doi:10.1016/j.celrep.2021.110196)
Supplement: 1 [file NIHMS1768918-supplement-1.pdf]

Cell Reports, Volume *38*

Supplemental information

Robust genome editing in adult vascular endothelium by nanoparticle delivery of CRISPR-Cas9 plasmid DNA

Xianming Zhang, Hua Jin, Xiaojia Huang, Birendra Chaurasiya, Daoyin Dong, Thomas P. Shanley, and You-Yang Zhao

SUPPLEMENTAL MATERIALS

Robust Genome Editing in Adult Vascular Endothelium

by Nanoparticle Delivery of CRISPR/Cas9 Plasmid DNA

Xianming Zhang, Hua Jin, Xiaojia Huang, Birendra Chaurasiya,
Daoyin Dong, Thomas P Shanley, and You-Yang Zhao

youyang.zhao@northwestern.edu

Supplemental Tables

Table S1. gRNA sequences against mouse *Pik3cg* or *Vegfr2*, Related to the Star Methods

Name	Sequence (5'→ 3')
Pik3cg-gRNA-1*	ACCGTACCACGACAGTGCGC
Pik3cg-gRNA-2	ATCTGGCCAGCGCACTGTCG
Pik3cg-gRNA-3	AGCCTCGCAGGTACGCCTCC
Pik3cg-gRNA-4	ACTAAAAGCCGGTACCCTGG
Vegfr2-gRNA-1	GTCCCGGTACGAGCACTTGT
Vegfr2-gRNA-2	TGATGTACACGATGCCATGC
Vegfr2-gRNA-3*	CAACCCTTCAGATTACTTGC
Vegfr2-gRNA-4	GAGCCTACAAGTGCTCGTAC

* indicates the potent gRNA

Table S2. QPCR primer sequences for genome editing analysis, Related to the Star Methods

Name	Forward (5'→3')	Reverse (5'→ 3')
Pik3cg-gRNA1	TTGAACCGTACCACGACAGTG	ACCAGAACAAGAAGTGACCGAT
Pik3cg-gRNA2	GAACTGTGGGTTTCCCCCAT	GAATCTGGCCAGCGCACTG
Pik3cg-gRNA3	TTGAACCGTACCACGACAGTG	AGCCTCGCAGGTACGCC
Pik3cg-gRNA4	TGGACTAAAAGCCGGTACCC	TGGTGCTAGTGATGAGAGGGT
Vefgr2-gRNA1	GGCGGTGGTGACAGTATCTT	CGTCCCGGTACGAGCACT
Vefgr2-gRNA2	GATGTCCGCATTCATGCAAGT	CGGTGATGTACACGATGCCA
Vefgr2-gRNA3	AGTGGAAATTGTTGTGACCTCAG	AGTGGAAATTGTTGTGACCTCAG
Vefgr2-gRNA4	ACTGGAGCCTACAAGTGCTCG	ACGACATTGGAAGCAGACGG

Table S3. Primer sequences for quantitative RT-PCR analysis of gene expression, Related to the Star Methods

Name	Forward (5' → 3')	Reverse (5' → 3')
Tnfa	CCCTCACACTCAGATCATCTTC	GTTTGCTACGACGTGGGCTACA
Il6	GGATACCACTCCCAACAGACC	CTGCAAGTGCATCATCGTTGT
Icam1	AGGGTGGCGGGAAAGTTCCTG	CGTCTGCAGGTCATCTTAGGAG
Ccna2	TGCAGCTGTCTCTTTACCCGCA	CTCCATTTCCCTAAGGTACGTG
Ccnb1	ACCAGAGGTGGAACTTGCTGA	ATGTTTCCATCGGGCTTGGAGA
Foxm1	CACTTGGATTGAGGACCACTT	GGTCGTTTCTGCTGTGATTCCA
Ppia	TCTTGTCCATGGCAAATGCTG	TGATCTTCTTGCTGGTCTTGC

Supplemental Figures and Figure Legends

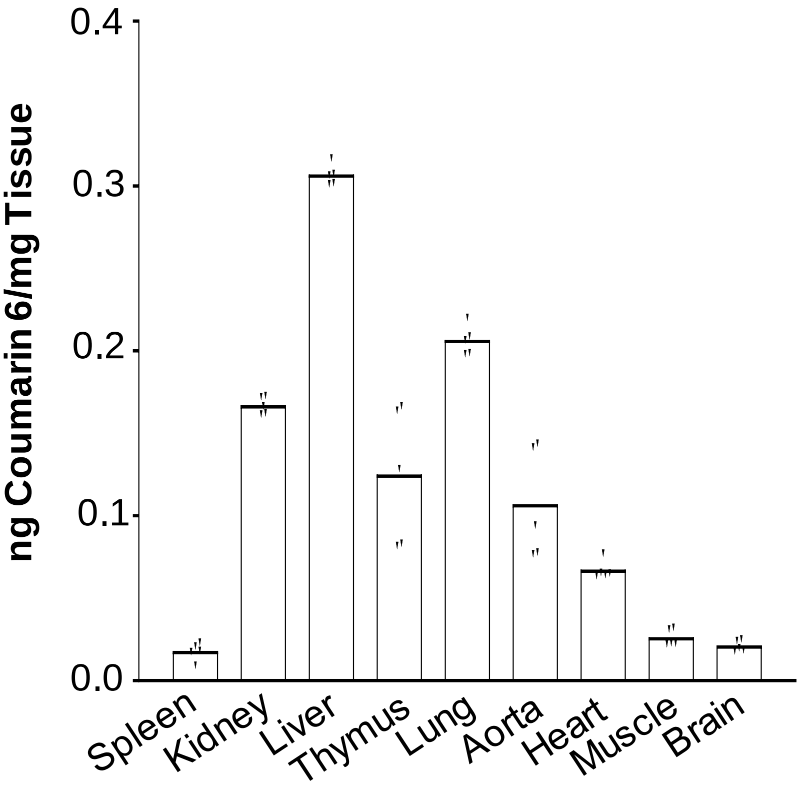

Figure S1. Biodistribution of PP/PEI nanoparticles. Related to Figure 1. Coumarin 6-loaded PP/PEI nanoparticles were administered to adult mice retro-orbitally. 8h later, various organ tissues were collected after bloodletting through abdominal aorta for homogenization and determination of Coumarin 6 fluorescent intensity. The amount of Coumarin 6 was calculated using a Coumarin 6 standard curve and normalized according to the tissue weight (ng/mg tissue) (n=5).

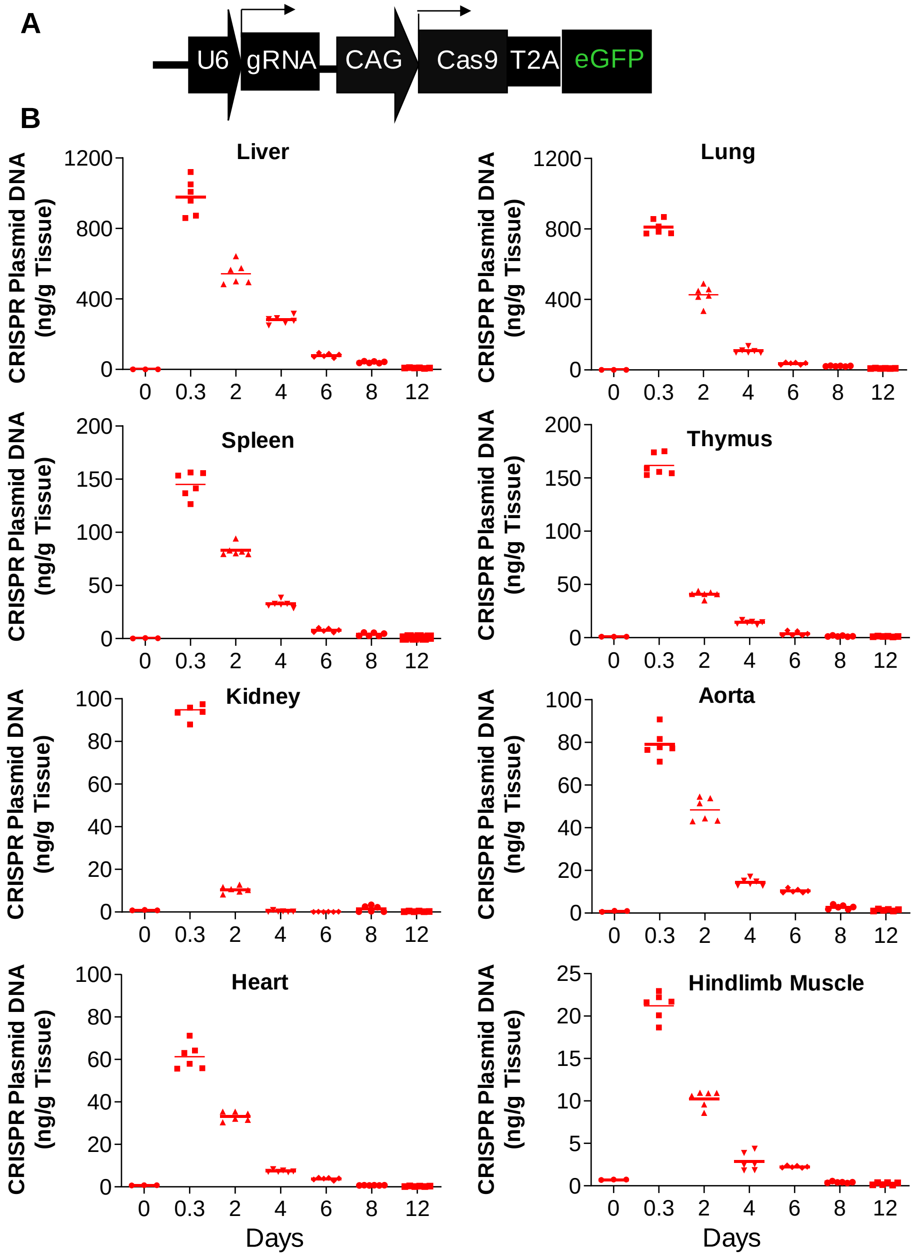

Figure S2. Pharmacokinetics of CRISPR plasmid DNA in various organs in adult mice. Related to Figure 1. (**A**) Schematic diagram of the all-in-one CRISPR^{CAG} plasmid DNA expressing Cas9 under the control of *CAG* promoter and gRNA driven by *U6* promoter. (**B**) Time course of plasmid DNA accumulation in various organs. The CRISPR plasmid DNA (40 µg/mouse) were delivered into adult mice by PP/PEI nanoparticles through retro-orbital injection. Tissues were collected after bloodletting through abdominal aorta at the indicated times after delivery. Plasmid DNA in each organ tissue was extracted after proteinase K digestion. The amount of Cas9 DNA was determined by QPCR analysis, and calculated using a standard curve generated from the CRISPR plasmid DNA. The amount of Cas9 DNA was normalized according to the tissue weight (ng/g tissue) (n=6). Bars represent means.

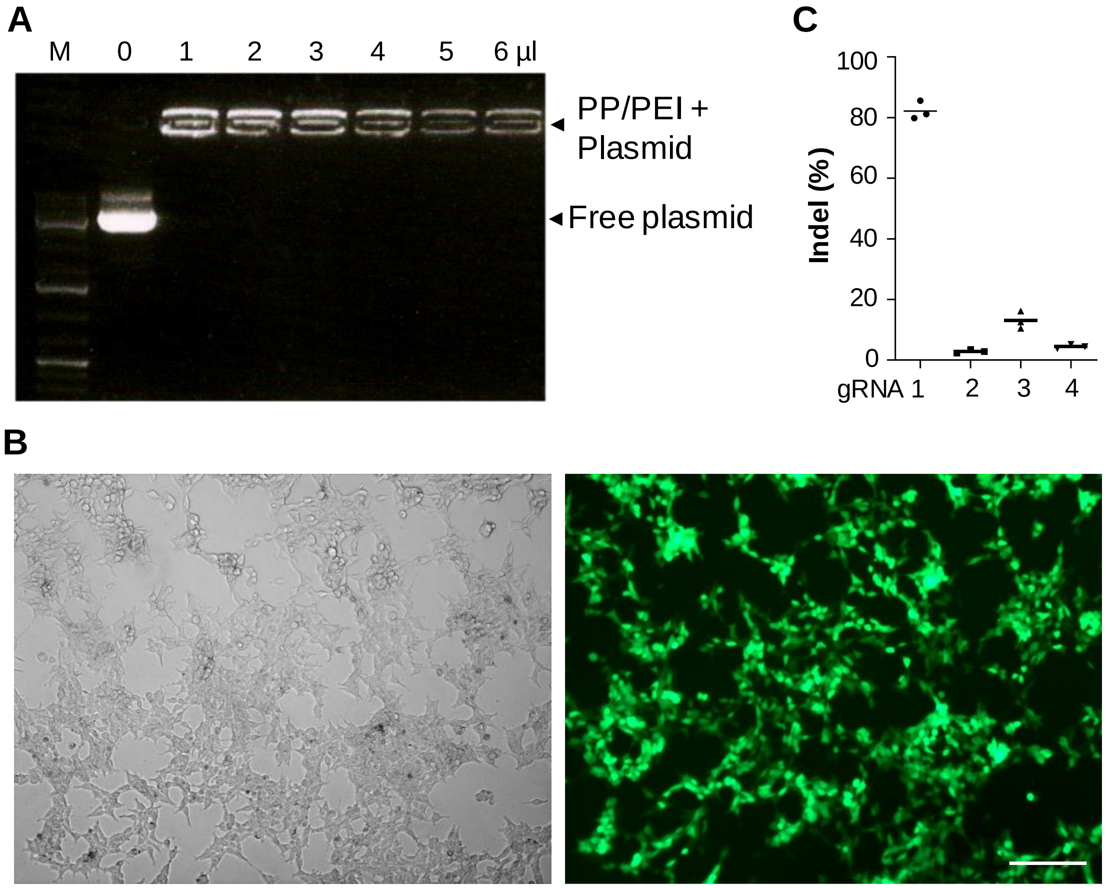

Figure S3. Identification of a potent *Pik3cg* gRNA in cultured cells. Related to Figure 2 and STAR Methods "In vitro Identification of potent gRNA…". (**A**) DNA electrophoresis demonstrating that PP/PEI nanoparticles neutralized the negative charge of CRISPR plasmid DNA and thus blocked their movement in the gel. 1 µg CRISPR^{CAG} plasmid DNA was mixed with various amount of PP/PEI (0-6 µl) nanoparticles and then loaded to 1% agarose gel for electrophoresis. M, molecular weight marker. (**B**) Representative micrographs of phase contrast and fluorescent microscopy demonstrating highly efficient transfection in cultured Hepa1c1c7 cells. Complexes of PP/PEI nanoparticle:CRISPR^{CAG} plasmid DNA were added to Hepa-1c1c7 cells for 48 h. Expression of GFP (green) was detected using fluorescent microscopy. Phase contrast image was shown to demonstrate the cell density. Scale bar, 100 µm. (**C**) Indel analysis identifying the potent gRNA. CRISPR^{CAG} plasmid DNA expressing gRNA specific for mouse *Pik3cg* gene was mixed with PP/PEI nanoparticles for 10 min at room temperature then transfected to subconfluent Hepa-1c1c7 cells. 48 h later, genomic DNA was extracted for Sanger sequencing decomposition analysis using TIDE software. gRNA1 is a potent gRNA which induced 80% genome editing efficiency (n=3).

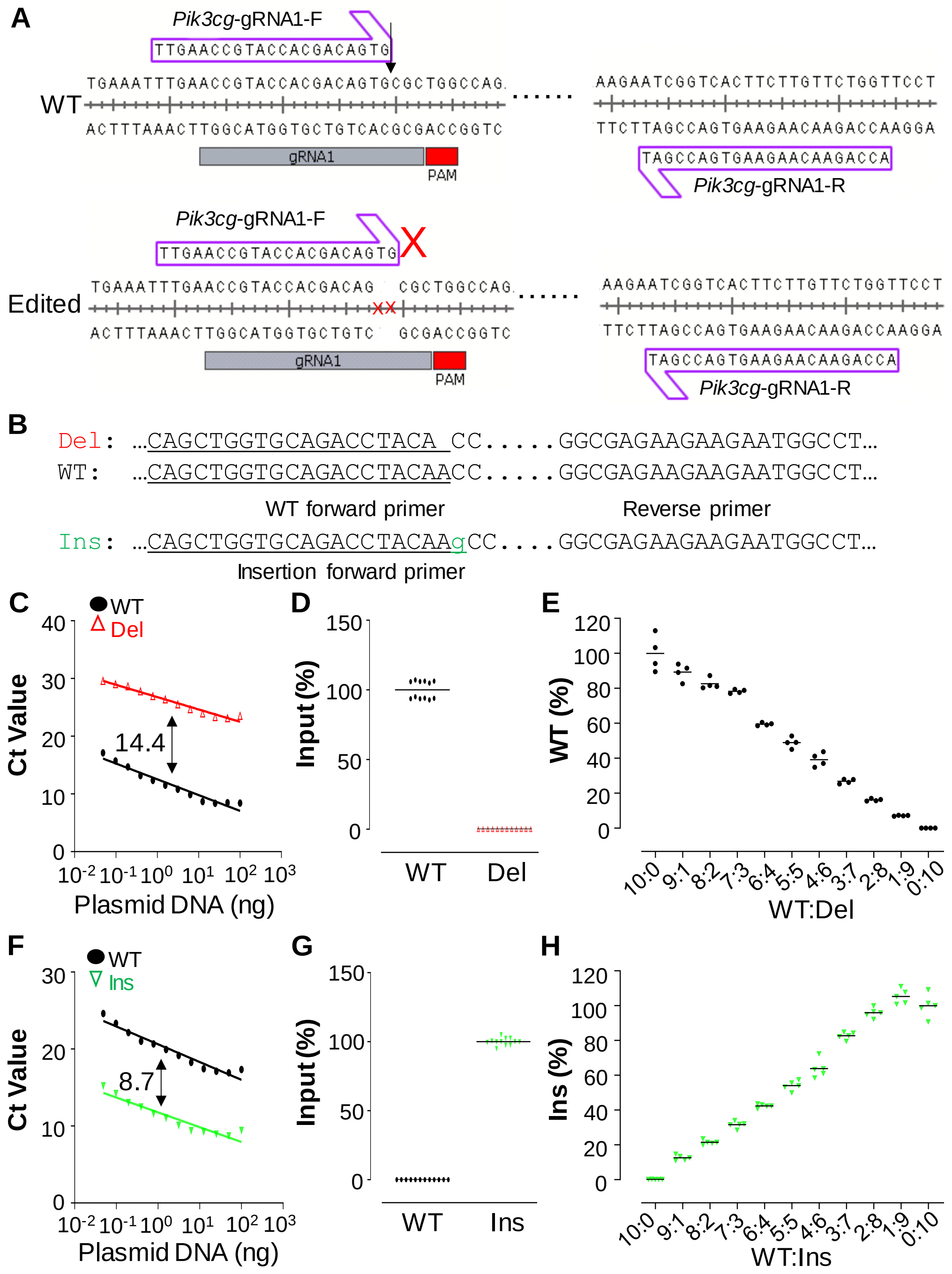

Figure S4. QPCR efficiently distinguished single base deletion or insertion from wild-type DNA. Related to Figure 2. (**A**) Schematic diagram of the strategy to design primers for QPCR analysis of indels. Arrow point to the predicted cleavage site. PAM, Protospacer Adjacent Motif sequence. (**B**) Diagram showing the single base deletion/insertion and the primers. (**C-E**) The WT forward primer could not amplify the fragment in plasmid DNA^{Del} with 1bp deletion (Del). (**F-H**) Quantitative detection of 1bp insertion in plasmid DNA^{Ins} with the Insertion forward primer which could not amplify the plasmid DNA^{WT} due to the 3' mismatch. n=12 (**D**, **G**), n=5 (**E, H**).

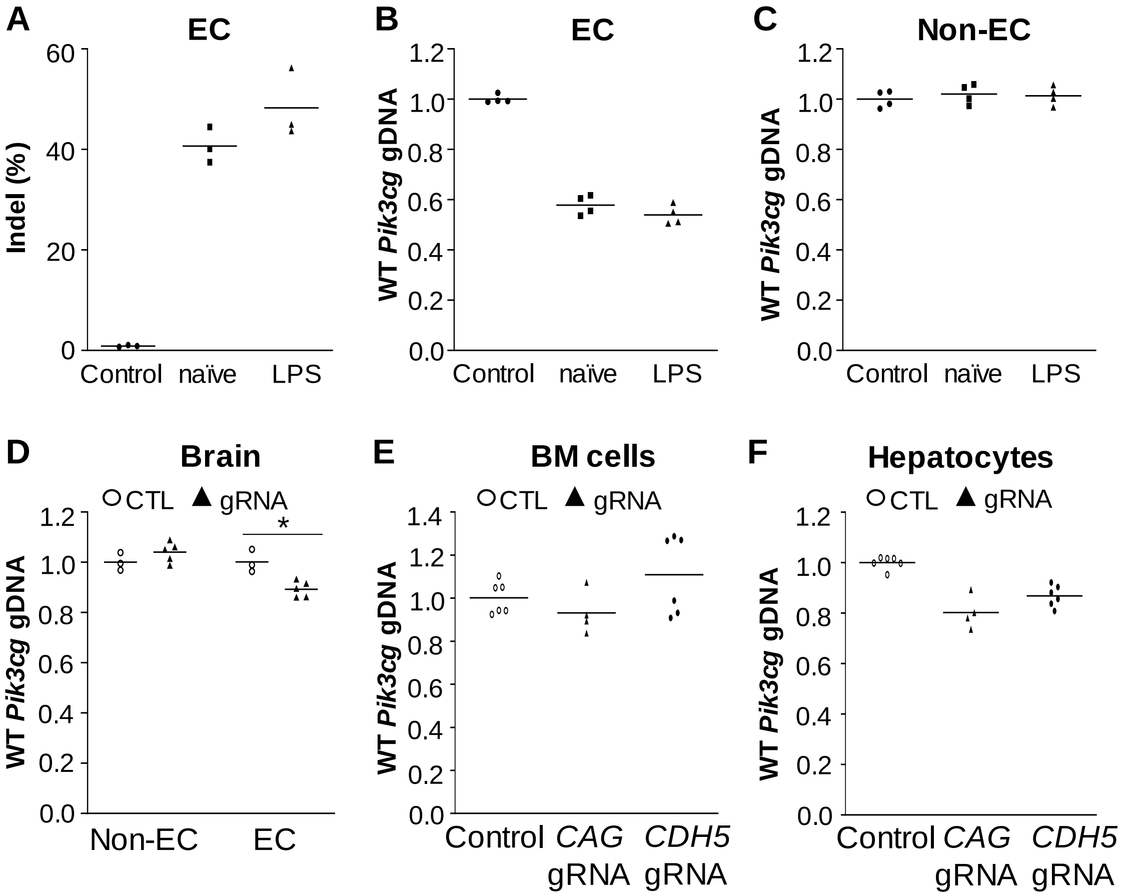

Figure S5. CRISPR^{CDH5} plasmid delivered by PP/PEI nanoparticles had comparable genome editing efficiency in lung ECs under conditions with and without inflammation. Related to Figure 3. (**A**) Next generation sequencing demonstrating similar efficiency of genome editing in lung ECs in naïve mice and septic mice. Same amount of CRISPR^{CDH5} plasmid DNA expressing gRNA1 (40µg/mouse) was delivered to wild-type mice at basal (naive) or 20h post-LPS challenge (2.5 mg/kg i.p.) (LPS) by retro-orbital injection with PP/PEI nanoparticles. Seven days after nanoparticle administration, lung tissues were collected for EC and non-EC isolation followed by indel analyses. Control, mice without nanoparticle delivery and LPS challenge (n=3). (**B**, **C**) QPCR analysis of wild-type (WT) genomic DNA of the genome editing region demonstrating similar efficiency of genome editing in lung ECs in naïve mice and septic mice (n=4). (**D-F**) Genome editing efficiency in brain ECs, bone marrow cells and hepatocytes in naïve mice. Small but significant genomic editing in cerebral vascular ECs (**D**). There was little genomic editing in bone marrow cells with either CAG or CDH5 promoter (**E**) whereas 20% genomic editing in hepatocytes in mice transduced the CRISPR^{CAG} plasmid DNA but not the CRISPR^{CDH5} plasmid DNA. n=3-6. * $P < 0.05$. Student's t test.

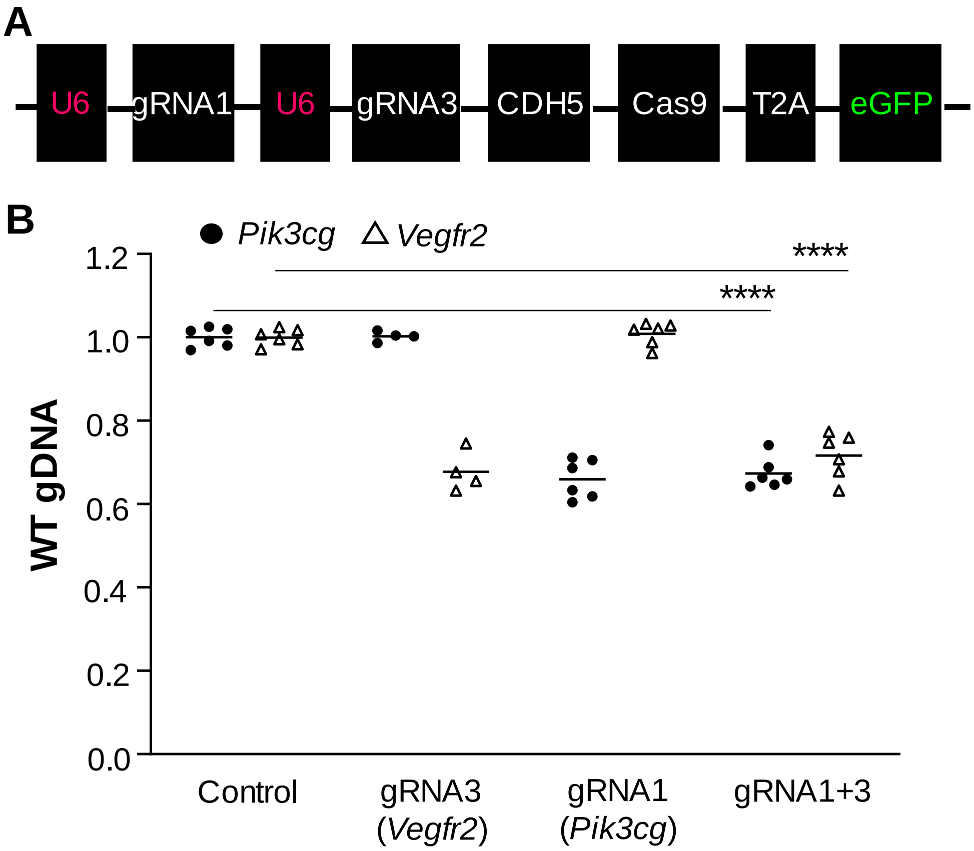

Figure S6. Simultaneous genomic editing of 2 genes in lung ECs with one plasmid DNA. Related to Figures 3 and 7. (**A**) Diagram presentation showing the CRISPRCDH5 plasmid expressing 2 gRNA against 2 different genes (gRNA1-*Pik3cg* and gRNA3-*Vegfr2*). (**B**) QPCR analysis showing similar efficiency of genome editing in lung ECs of mice transduced the CRSIPR plasmid DNA expressing single gRNA or 2 gRNAs. Each mouse was delivered 40 µg plasmid DNA by PP/PEI nanoparticles and lung tissues were collected at 7 days later for EC isolation followed by QPCR analysis with wild-type primers for Pik3cg and Vegfr2, respectively (n=4 or 6). **** $P < 0.0001$. Student's *t* test.

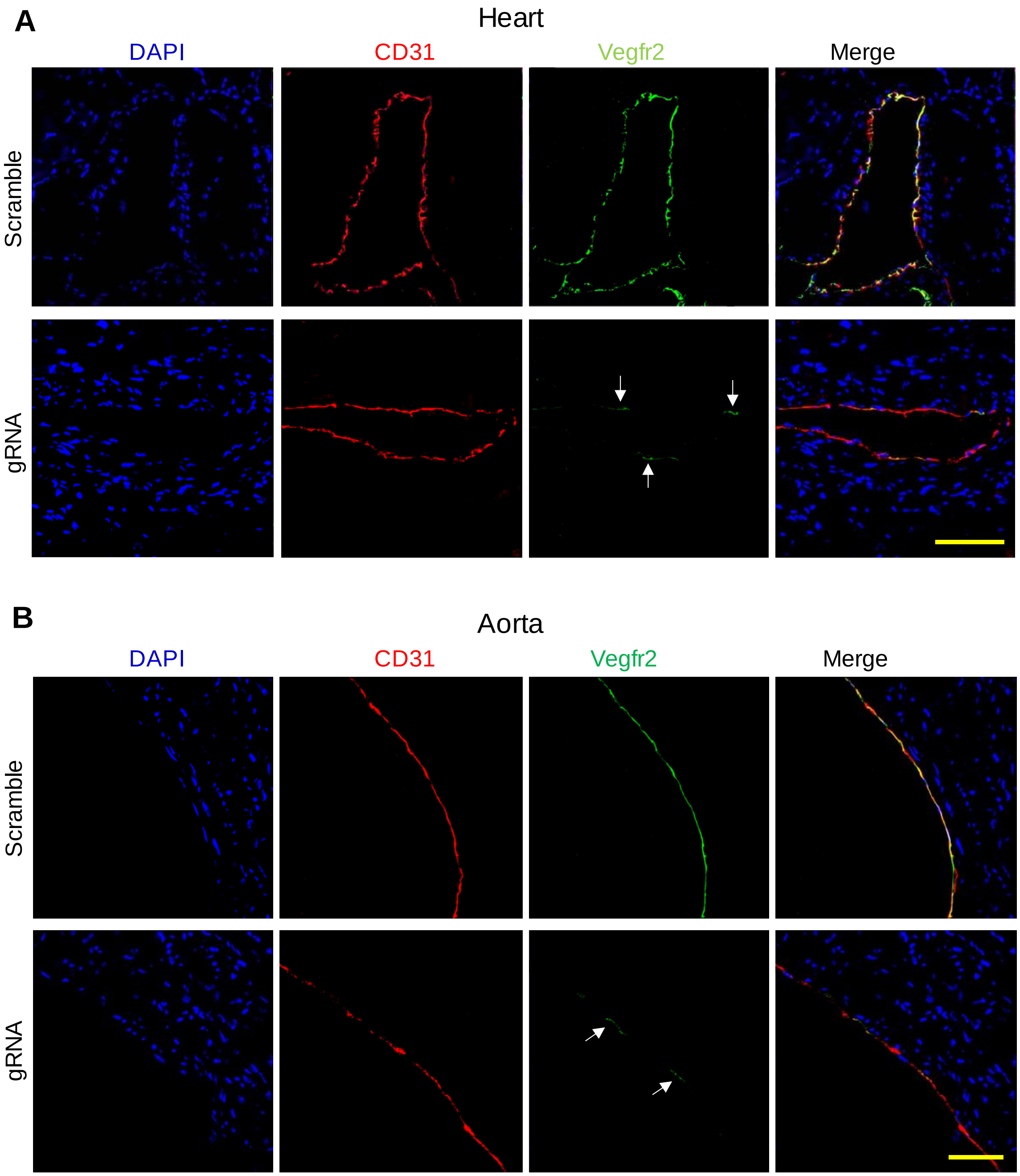

Figure S7. Representative micrographs of immunostaining showing diminished Vegfr2 expression *Vegfr2* gRNA-CRISPR^{CDH5} plasmid DNA-transduced mice. Related to Figure 7. A mixture of PP/PEI nanoparticles:CRISPR^{CDH5} plasmid DNA expressing *Vegfr2* gRNA or scramble RNA was administered to adult WT mice (40µg/mouse) retro-orbitally. 7 days later, heart and aorta tissues were collected for cryosectioning and immunostaining with anti-Vegfr2 (green) and anti-CD31 (red). Nuclei were counter-stained with DAPI (blue). Arrows point to ECs with less efficient knockdown of Vegfr2. Scale bars, 50µm.
